# Pathogenicity and virulence regulation of *Vibrio cholerae* at the interface of host-gut microbiome interactions

**DOI:** 10.1080/21505594.2020.1845039

**Published:** 2020-11-11

**Authors:** Ansel Hsiao, Jun Zhu

**Affiliations:** ^a^Department of Microbiology & Plant Pathology, University of California Riverside, Riverside, CA, USA; ^b^Department of Microbiology, Perelman School of Medicine, University of Pennsylvania, Philadelphia, PA, USA

**Keywords:** *Vibrio cholerae*, virulence factors, microbiome, quorum sensing, bile salts, anaerobiosis, small intestine, T6SS

## Abstract

The Gram-negative bacterium *Vibrio cholerae* is responsible for the severe diarrheal pandemic disease cholera, representing a major global public health concern. This pathogen transitions from aquatic reservoirs into epidemics in human populations, and has evolved numerous mechanisms to sense this transition in order to appropriately regulate its gene expression for infection. At the intersection of pathogen and host in the gastrointestinal tract lies the community of native gut microbes, the gut microbiome. It is increasingly clear that the diversity of species and biochemical activities within the gut microbiome represents a driver of infection outcome, through their ability to manipulate the signals used by *V. cholerae* to regulate virulence and fitness *in vivo*. A better mechanistic understanding of how commensal microbial action interacts with *V. cholerae* pathogenesis may lead to novel prophylactic and therapeutic interventions for cholera. Here, we review a subset of this burgeoning field of research.

## Introduction

*Vibrio cholerae* is a Gram-negative bacterium responsible for the severe human diarrheal disease cholera. Cholera is characterized by voluminous watery diarrheas and vomiting, which may rapidly lead to hypovolemic shock, acidosis, and death, with an untreated case-fatality rate as high as 50%. Seven recorded major cholera pandemics have been recorded since 1871, though the disease likely has an ancient association with humans. Although the development of oral rehydration has reduced the treated case fatality rate substantially, cholera continues to impose an enormous global health burden. One contributor to this is the high morbidity of cholera; though patients are likely to survive infection due to rehydration therapy, the debilitating diarrhea characteristic of cholera continues for days even if the pathogen is cleared with antibiotics. Cholera is widely distributed, and represents a threat to public health in many parts of Asia, Africa, and Latin America. Each year sees 1.3–4.0 million cases of cholera, and 21,000–143,000 deaths worldwide, as estimated by the World Health Organization (WHO) [1]. Therefore, infection with *V. cholerae* remains an important health and economic concern [2–5]; the Roadmap to 2030 from the WHO Global Task Force on Cholera Control (GTFCC) envisages a plan for a 90% reduction in deaths from cholera [6].

Although *V. cholerae* is serologically diverse with more than 206 serogroups reported, only O1 and O139 serogroups have been known to cause epidemic cholera [2]. The first six cholera pandemics were caused by classical biotype *V. cholerae*, whereas the El Tor biotype is responsible for the current, seventh pandemic. These two *V. cholerae* biotypes differ considerably; El Tor strains generally cause a milder form of cholera than that caused by classical strains and apparently evolved as better survivors in the aquatic environment [7]. Currently, El Tor strains are predominant everywhere that *V. cholerae* O1 can be found [2].

Between epidemics, *V. cholerae* natively resides in aquatic environments such as freshwater lakes and rivers, where these bacteria interact with various surfaces in the form of biofilms, which form an important survival mechanism [8]. From these aquatic reservoirs, *V. cholerae* spreads to populations of the natural host, humans, through contamination of water and food. Upon human colonization, virulence is due primarily to the production of cholera toxin (CT), which alters host cell signal transduction pathways and leads to cell damage and diarrhea, and the toxin coregulated pilus (TCP), which is critical for colonization of the intestinal epithelium [9]. The profuse watery diarrhea of cholera then disseminates the pathogen back into the environment, where the cycle of fecal contamination can continue, leading to epidemic spread in human populations.

*V. cholerae* is able to respond to environmental signals in the transition from the aquatic environment into the gastrointestinal tract, and regulate virulence genes coordinately to allow for colonization, survival, virulence, and subsequent broad dissemination. The environmental conditions of the gut are tightly bound to the activities of the native microbial community of the gastrointestinal tract, the gut microbiome. Due to the rapidly expanding field of research, this review will focus on just a subset of recent work on how *V. cholerae* adapts to *in vivo* environments by responding to intestinal signals produced and modulated by the host and by commensal microbes.

## Regulatory networks to coordinately activate virulence genes during infection

A body of work on the ecology of cholera has demonstrated that *V. cholerae* is an autochthonous aquatic organism, occupying brackish water and estuarine environments when not associated with the human host [10,11], underlined by the presence of multiple adaptive strategies for persistence in aquatic reservoirs. Outside of the human host, *V. cholerae* can be found complexed with marine organisms such as crustacean zooplankton [12,13]. *V. cholerae* have been found as biofilms on zooplankton host exoskeleton, as well as in the gut of these marine organisms. Planktonic *V. cholerae* can also respond to low temperatures or nutrient limitation by phenotypic transition to a viable but non-culturable (VBNC) state [14] characterized by reduced cell size and minimal metabolic activity. These environmental persistence strategies have major consequences for the transition into the host gastrointestinal tract. Biofilm-associated *V. cholerae* is more acid-resistant than planktonic cells, which aids in the transition through the low-pH environment of the stomach and ultimately into the distal small intestine, the preferred site of human colonization [15]. There, *V. cholerae* also needs to exit biofilm structures in order to disseminate into the gut mucosa. This process is aided by gut-localized environmental factors such as bile [16].

As they enter the host, *V. cholerae* cells are exposed to a series of changes, such as temperature, osmolarity, oxygen concentration, and exposure of antimicrobial agents (e.g. bile salts). Given the dramatically different environmental conditions between the aquatic reservoir and the host mucosa, it is unsurprising that *V. cholerae* have evolved numerous regulatory mechanisms designed to tailor the production of factors permitting optimal host colonization.

The ability of *V. cholerae* to colonize and cause disease in hosts requires production of a number of virulence factors during infection. The two major virulence determinants of *V. cholerae* are encoded by two separate genetic elements. Cholera toxin, which causes the diarrhea characteristic of cholera, is encoded by *ctxAB* genes on the lysogenic CTXΦ bacteriophage [17]. *V. cholerae* also produces toxin-coregulated pili (TCP), which are required for intestinal colonization both in animal models and in human volunteers [18,19]. TCP is thought to be a polymer of the main structural subunit, TcpA, and serves as the receptor for the CTXΦ bacteriophage [17,20]. The genes required for TCP synthesis, including *tcpA* as well as accessory colonization factor (*acf*) genes and the genes encoding the virulence transcriptional activators ToxT and TcpP, are located on a 40-kb *Vibrio* pathogenicity island [21]. Coordinate expression of *V. cholerae* virulence genes results from the activity of a cascading system of regulatory factors [22].

The primary direct transcriptional activator of *V. cholerae* virulence genes, including *ctxAB* and *tcpA*, is ToxT, a member of the AraC/XylS-family of transcriptional regulators [23], members of which often bind effector molecules and/or oligomerize to affect transcriptional regulation at target promoters [24]. Unsaturated fatty acids can directly bind to and inhibit the activity of ToxT [25–27]. To activate transcription, ToxT recognizes and binds to a degenerate 13-bp DNA sequence, the “toxbox”[28]. FadR, the master regulator of fatty acid metabolism, modulates ToxT activity at both transcriptional and posttranslational levels [29], suggesting that the expression of genes involved in fatty acid biosynthesis and virulence are intertwined.

**Figure 1.**
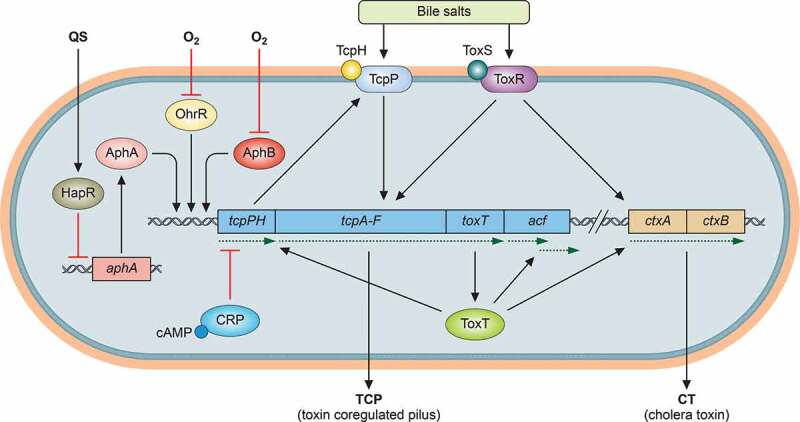
*V. cholerae* virulence regulatory networks. Major transcriptional regulators and their corresponding signals are shown. In particular, the master regulator ToxT activates virulence genes which products are involved in synthesis of the key virulence determinants TCP and CT. The expression of toxT is regulated by TcpP and ToxR. QS: quorum sensing. →: activation; ┴: repression

A complex regulatory pathway controls the expression of ToxT (Figure 1). The ToxR protein was identified as the first positive regulator of *V. cholerae* virulence genes; the genes involved in the transcriptional cascade resulting in *toxT* expression and consequent virulence gene activation is thus often referred to as the “ToxR regulon”[30]. Acting in conjunction with TcpP (see below), ToxR activates the transcription of *toxT* [31–33]. ToxR directly regulates the transcription of many genes [34], including *ompU* and *ompT*, which encode the major porins of *V. cholerae* [35,36]. ToxR is a bitopic membrane protein containing a cytoplasmic DNA-binding domain, a single transmembrane domain, and a periplasmic domain. ToxR activity requires the presence of another inner membrane protein, ToxS; deletion of *toxS* negatively impacts ToxR transcriptional activity [37], suggesting that ToxS serves as an effector of ToxR function by influencing the stability and/or the dimerization of ToxR [23,38,39]. Recent work has also shown that ToxR-ToxR protein–protein interactions are significantly increased in response to ToxR operators and the co-activator ToxS [40].

To activate the expression of *toxT*, ToxR acts with a second transcription activator, TcpP, which is also membrane-localized and contains a cytoplasmic winged helix-turn-helix (w-HTH) domain [32]. TcpP, like ToxR, requires the presence of a membrane-bound effector protein, TcpH, which interacts with TcpP [41]. TcpP is degraded by a protease in the absence of TcpH, and during conditions unfavorable for virulence gene activation [42,43]. TcpP binds to the *toxT* promoter just upstream of the −35 element and is a direct *toxT* activator [44]. Overexpression of TcpP alone activates *toxT* expression [45,46], but binding of ToxR to the upstream of TcpP-binding site is required for TcpP-mediated expression of *toxT* at endogenous expression levels [47,48].

Two activators encoded by unlinked genes, AphA and AphB, regulate transcription of *tcpPH*. AphA is a dimer with an N-terminal winged-helix DNA binding domain that is structurally similar to those of MarR family transcriptional regulators [49]. AphA cannot activate transcription of *tcpPH* alone, but requires interaction with the LysR-type regulator AphB that binds downstream of AphA binding site [50]. This interaction is thought to stabilize AphB binding to its recognition site and result in activation of the *tcpPH* promoter. In addition, AphB enhances the expression of *toxR* [51]. Expression of *aphA* is also controlled by a quorum-sensing system [50,52–54], discussed in detail below. This process means that virulence gene expression declines at high cell density and is thought to contribute to the self-limiting nature of *V. cholerae* infections.

## Gastrointestinal signals modulate virulence

Pathogens that cause diseases of complex animal hosts require clever strategies for survival and multiplication during the dynamic conditions found during infection. Often pathogens take advantage of host-specific signals to modulate their gene expression in order to adapt the new environments. *V. cholerae* encounters a variety of unique host-specific signals, including bile, differences in osmolarity, oxygen availability, changes in pH as it travels from the aquatic reservoir to the stomach and into the intestines. Ample research has demonstrated that this bacterium has evolved to rapidly respond to these signals to promote survival and proliferation in the gut, and below we review a subset of these *in vivo* signals.

### Bile

Bile is a digestive secretion primarily involved in emulsifying and solubilizing dietary lipids to aid absorption, and is composed of bile acids, cholesterol, phospholipids, and IgA [55]. Bile acids, the predominant component of bile, are synthesized in the liver from cholesterol as primary bile acids, often conjugated to amino acids such as taurine and glycine. Bile is stored in the gall bladder, to be secreted into the small intestine with the intake of food, where due to the local pH they are often found as primary bile salts. In the intestines, bile acids/salts mediates digestive processes, and are modified by the action of gut microbes into several secondary bile molecules [56–58]. Up to 95% of bile acids are reabsorbed from the distal ileum, to be passed back to the liver via portal circulation to be re-conjugated to amino acids and re-secreted in a process called enterohepatic circulation [59,60].

Bile can destabilize membranes and disrupt bacterial cellular homeostasis via its detergent-like properties. As a result, the large quantity of bile secreted by liver every day represents a challenge for invading pathogens [61]. *V. cholerae* is highly resistant to bile through the action of efflux pumps and by outer membrane porins selectively restricting the influx of bile salts [62–65]. The compartment-specific cycling of bile acids means that these molecules can serve as a convenient spatiotemporal cue for gene regulation in microbes adapted to the small intestine. Bile has been shown to promote *V. cholerae* motility, which is required for efficient colonization [66,67]. Most prominently, *V. cholerae* is able to use a set of largely primary bile salts (taurocholate, glycocholate) to activate virulence gene expression [68]. This activation is mediated through the transmembrane transcription factor TcpP. Various genetic and biochemical analyses indicate that in the absence of bile salts, one of the two cysteine residues in the periplasmic domain, Cys^218^, forms an inhibitory intramolecular disulfide bond with the other cysteine residue Cys^207^. Taurocholate promotes the formation of C^207^-C^207^ intermolecular disulfide bond formation of TcpP and dimerization, and thus increasing the activity TcpP. Further investigation [69] showed that bile salts inhibit the reductase activity of DsbA, a conserved oxidoreductase in the bacterial periplasm that participates in protein folding by introducing disulfide bonds into proteins [70]; indeed, DsbA induces TcpP dimerization in the presence of primary bile salts such as taurocholate. Calcium has been shown to enhance virulence by promoting bile salt-induced TcpP–TcpP interaction [71]. Bile salts can also prevent ToxR proteolysis and promote ToxR-ToxR protein interaction and ToxRS complex formation [40,72]. One bile salt, taurocholate, promotes *V. cholerae* dispersal from biofilm structure [16], in addition to activating virulence genes, suggesting that if *V. cholerae* is ingested as a biofilm to protect against reduced stomach pH, it has coopted the host-derived bile salt signal to detach from the biofilm and go on to activate virulence. Bile is a highly complex mixture and its components have been reported to have different effects on *V. cholerae* infection; as mentioned above, unsaturated fatty acids that are found in bile bind to ToxT and inhibit its transcriptional activity [25]. Crude bile has been found to decrease cholera toxin production, and fatty acids with bile can repress other aspects of *V. cholerae* virulence through ToxT [25,73].

### Anaerobiosis

Given the presumed low oxygen concentration of the gut [74], it is unsurprising that anaerobiosis serves as one of the host environmental factors that modulate virulence factor production [75]. *V. cholerae* encounters low oxygen concentrations in the upper intestine, and transcriptional examinations of *in vivo* grown bacteria have confirmed the expression of metabolic genes responsible for anaerobic energy metabolism [76]. Anaerobic respiration of trimethylamine N-oxide (TMAO) enhance cholera toxin production, and TMAO induces more severe symptoms in the infant mouse model [77]. Oxygen availability has been shown to modulate virulence gene expression in a number of gastrointestinal pathogens, such as *Shigella* [78], *Salmonella* [79,80], and enterohaemorrhagic *E. coli* [81]. Under anaerobic conditions, *tcpP* expression increases, and work has shown that this effect acts through AphB [82]; anaerobiosis enhances oligomerization and activity of AphB [83]. Specifically, one key AphB cysteine residue (Cys^235^) is oxidized under aerobic conditions. Under low oxygen conditions, AphB Cys^235^ is reduced, which promotes oligomerization and subsequently enhances AphB activity. Intriguingly, during the transition from oxygen-rich to oxygen-poor environments, the rate of reduction of AphB is slow. Another redox-sensing regulator, OhrR, whose cysteine reduction is faster than that of AphB, is needed to jump-start virulence gene expression [84]. Anaerobiosis also promotes ToxR-TcpP interaction, which is important for virulence gene induction [85]. Other global regulatory systems for anaerobic metabolism, such as the ArcA/ArcB two-component system, may also be involved in modulating expression of *V. cholerae* virulence genes [86], though the exact mechanism of this regulation is unclear.

### Catabolite sensing and nucleotide-derived second messengers

In recent years, it has become more and more apparent that nucleotide-derived small molecules that accumulate intracellularly are important for bacterial environmental adaptation. These so-called second messengers often have global regulatory effects across bacterial species [87]. In *V. cholerae*, these small molecules control important physiological functions such as virulence and biofilm formation. Among them, the well-studied cyclic adenosine monophosphate (cAMP) is primarily associated with the regulation of carbon utilization. The cAMP regulation is mediated through the cyclic AMP (cAMP) receptor protein (CRP), which binds DNA in response to the intracellular availability of cAMP. CRP-cAMP negatively regulates virulence genes, including cholera toxin genes, TCP pilin gene *tcpA*, and *tcpP* [88,89]. Recently, the Camilli group used chromatin immunoprecipitation coupled with DNA sequencing (ChIP-seq) to map the distribution of CRP binding sites across the *V. cholerae* genome [90] and found that CRP-regulated genes substantially overlaps the ToxR regulon, and that CRP also controls additional virulence factors not regulated by ToxR, such as production of RTX toxin. Another well-studied molecule is guanosine penta/tetraphosphate ((p)ppGpp), which is a primary signal in the bacterial stringent response [91]. In *V. cholerae*, ((p)ppGpp) signaling pathways regulates antibiotic tolerance [92]. In recent years, the importance of cyclic dinucleotide second messengers has become widely appreciated. Bis (3ʹ-5ʹ) cyclic dimeric guanosine monophosphate (c-di- GMP) has been found in all major bacterial phyla. C-di-GMP is produced by diguanylate cyclases and degraded by phosphodiesterases [93]. The *V. cholerae* genome contains 42 genes encoding putative diguanylate cyclases/phosphodiesterases [94]. In *V. cholerae*, c-di-GMP enhances biofilm matrix production and represses motility as well as virulence gene expression [95–101], suggesting that c-di-GMP may be regulated in response to a variety of environmental signals. Additionally, the most recently identified bacterial cyclic dinucleotide signaling molecule, the hybrid 3′, 3′-cyclic GMP-AMP (cGAMP), is synthesized by the enzyme DncV, whose expression is regulated by the master virulence regulator ToxT [102]. cGAMP binds to the effector called CapV, a phospholipase, and regulates virulence, chemotaxis, and fatty acid metabolic genes [103].

### ROS/RNS

One of the major stresses in the intestine that *V. cholerae* must overcome are those generated by exposure to reactive radical species. Reactive compounds, including reactive oxygen species (ROS) and reactive nitrogen species (RNS), are produced in the gut during *V. cholerae* infection [104–106]. Several proteins have been identified in *V. cholerae* ROS resistance, including catalases (KatG and KatB), peroxiredoxin (PrxA), organic hydroperoxide resistance protein (OhrA), a redox-regulated chaperone (Hsp33), and a DNA-binding protein from starved cells (DPS) [107–110]. ROS resistance in *V. cholerae* is tightly regulated by a variety of mechanisms. OxyR is required to activate catalase genes and *dps*, and is modulated by another OxyR homolog, OxyR2 [107,109,111]. Quorum sensing systems [54], PhoB/PhoR two-component systems [112], and the virulence regulator AphB also play important roles in the *V. cholerae* oxidative stress response [113]. Interestingly, ROS may also enhance the *V. cholerae* mutation rate *in vivo*, which results in increased catalase production and increased biofilm formation, leading to colonization advantages in ROS-rich intestines [114]. Adaptive responses in many bacterial pathogens are induced by nitric oxide and RNS. For example, in an inflamed gut, NO is converted to nitrate, which is used by enteric pathogens, such as *S*. Typhimurium and *E. coli*, as an anaerobic respiration substrate, leading to pathogen expansion at the expense of the native gut microbiota [115–118]. *V. cholerae* lacks a nitrite reductase, but nitrate reduction in *V. cholerae* can occur at alkaline pH during hypoxic growth, whereas in acidic conditions, accumulation of NO_2_ from NO_3_ simultaneously limits growth while preserving viability [119]. It has also been shown that a NO-activated transcriptional regulator, NorR, positively regulates *hmpA*, which encodes a flavohemoglobin that is critical for *V. cholerae* RNS resistance *in vitro* and *in vivo* [120,121].

## Temporal gene expression during *V. cholerae* infection

**Figure 2.**
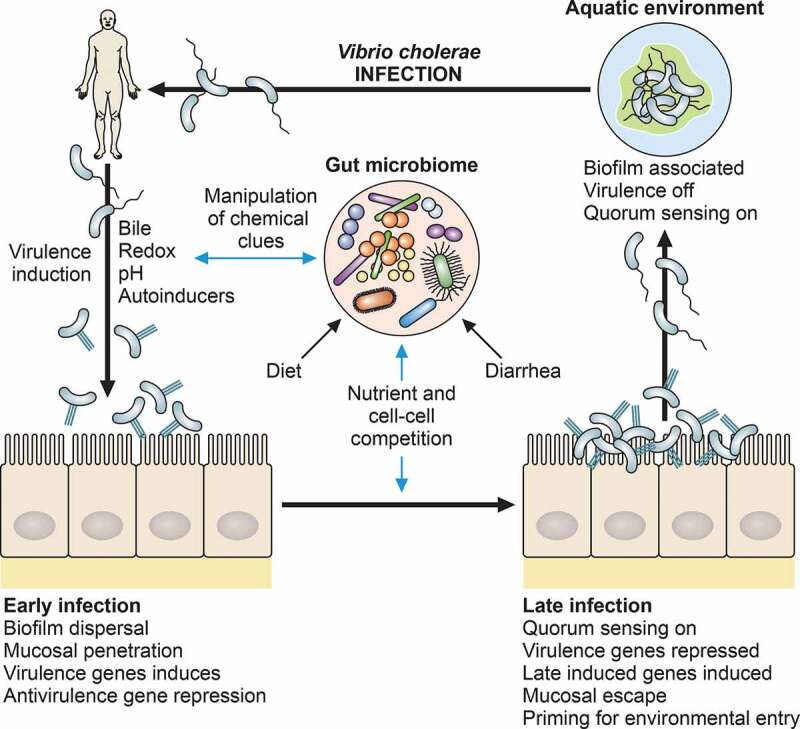
Interaction of the gut microbiome with environmental signaling during *V. cholerae* life cycle. The microbiome is shaped on an individual basis by diet, microbial exposure, and history of gut insults such as diarrhea, malnutrition, and inflammation. Commensal microbial functions influence chemical cues used by *V. cholerae* to time gene expression during early vs late infection states

Facultative bacterial pathogens such as *V. cholerae* that transit from environmental reservoirs to host populations and back are rarely in a static environmental context. Thus, these bacteria must continuously adapt, re-tooling their transcriptional and translational repertoires in order to suit the varied environments they encounter. For example, toxins, host-specific adherence/attachment and invasion factors, loosely classified under the designation “virulence genes,” are activated in an infection-specific manner, whereas the expression of some genes important for environmental survival are down-regulated during infection in order to evade host defense mechanisms (Figure 2).

### In vivo induced genes

Very often virulence genes are only highly expressed *in vivo; in vitro* conditions developed to induce virulence gene expression may thus seem artificial. For instance, the laboratory conditions required for activation of TCP and CT in the classical biotype (30°C, pH 6.5, and low osmolarity) are obviously different from the conditions in the small intestine [122]. In the El Tor biotype, one artificial condition (AKI) has been defined that induces expression of CT and TCP [123]. In an elegant study [124], Camilli and colleagues used a recombination-based *in vivo* expression technology (RIVET) to study *in vivo* timing of gene induction at the single-cell level. They found that while cholera toxin gene is induced monophasically in the small intestine, the expression of *tcpA*, which encodes the major subunit of TCP, is induced biphasically in two temporally and spatially separable events during *V. cholerae* infection. Intriguingly, ToxR, TcpP, and ToxT are all required to activate virulence genes *in vitro* but only ToxT is fully required *in vivo*. Later RNA-Seq analysis identified that transcripts elevated in infected rabbits and mice relative to laboratory media are regulated not only by previously identified regulators, but also by genes and small RNAs previously not linked to virulence [125], suggesting that virulence activation *in vivo* is more complex than previously thought.

### In vivo repressed genes

Pathogenic bacteria must contend with an *in vivo* environment that is under the protection of host mechanisms capable of rapidly identifying and eliminating foreign microorganisms. Successful pathogens often have mechanisms to survive the recognition by components of the host immune system. Using a differential fluorescence-activation approach, Hsiao et al. reported [126] that among the genes that are repressed by *V. cholerae* during infection are those encoding for the biogenesis of a Type IV mannose-sensitive hemagglutinin (MSHA), a structure that is produced *in vitro* and is important for *V. cholerae* biofilm formation [127], but not during early colonization of the infant mouse model. This is an important process for *V. cholerae* during infection, as host-secreted IgA nonspecifically binds to *V. cholerae* cells in an MSHA-dependent manner. Bacteria bound by secretory immunoglobulin in the gut can become entrapped in the mucus layer of the intestine, excluding them from the epithelium and leading to clearance by bulk flow [128–130]. Interestingly, it has been shown that the transcription factor responsible for the negative regulation of *msh* gene transcription is ToxT, the same transcription factor responsible for direct virulence gene induction [131]. Thus, this pathogen very efficiently combines its responses to host stimuli in order to simultaneously upregulated factors necessary for colonization (*ctx* and *tcp* genes), while using these same factors to transcriptionally repress the biogenesis of anti-colonization factors such as MSHA. Recently, another study used a different approach to identify *in vivo*-repressed genes in *V. cholerae* and found that *clcA*, encoding an H^+^/Cl^−^ transporter, is repressed during infection [132]. While ClcA facilitates survival under low pH (e.g., the stomach), its activity becomes detrimental under the alkaline conditions found in the small intestine; indeed, constitutive expression of *clcA* reduces colonization fitness.

### Late induced genes

Schild and colleagues employed a modified the *tnpR* RIVET system described above [124,133] to screen for genes only induced late in infection [134]. The library of promoters can be culled at any given point during infection by the oral administration of kanamycin to infected animals, which kills those cells that have already expressed *tnpR* and resolved the *neo-sacB* cassette. Interestingly, many of the genes found to have been induced in later stages of infection are involved in bacterial metabolism, indicating that *V. cholerae* initiates a transcriptional program to prepare for life outside the host. Using a host-to-environment transition assay, these genes are shown to be important for *V. cholerae* to persist within cholera stool and/or aquatic environments. In addition, biogenesis genes required for the environmental adhesin, MSHA pili is also upregulated, further suggesting that *V. cholerae* are transcriptionally committing to life outside of the host during exit.

### Quorum sensing regulates *V. cholerae* transmission and dissemination

Quorum sensing (QS) refers to the phenomenon in which bacteria produce and exchange chemical signals to monitor population density [135–138]. Many Gram-positive and Gram-negative bacteria use quorum sensing to control a variety of physiological functions. *V. cholerae* QS regulatory systems are highly complex. At low cell densities, the components of the QS pathway act as kinases to phosphorylate LuxO, which in turn activates the transcription of small RNAs (*qrr1-qrr4*) that destabilize mRNA of *hapR*, encoding the QS master regulator [139] and activate *aphA*, encoding the virulence regulator. At high cell densities, two sets of autoinducers, CAI-1 ((S)-3-hydroxytridecan-4-one) and AI-2 ((2S,4S)-2-methyl-2,3,3,4-tetrahydroxytetrahydrofuran borate) [140–142], bind to cognate sensors on the bacterial surface and induce conformational changes in the sensors [143], which results in dephosphorylation of LuxO. Thus, expression of the Qrr sRNAs is repressed by AI-2 and CAI-1. In addition, two other receptor proteins, CqsR and VpsS, have been reported to channel information through LuxO [144]. More recently, Recently, Bassler’s group has discovered another QS system [145] that does not require LuxO or the Qrr sRNAs, but rather relies on another autoinducer, DPO (3,5-dimethylpyrazin-2-ol), which is synthesized by the threonine dehydrogenase Tdh. DPO is sensed by VqmA, a LuxR-type transcriptional regulator [146], which induces the transcription of the VqmR sRNA. VqmR inhibits transcription of multiple target, including *vpsT* [97,147] that encodes a key activator of biofilm formation, as well as the virulence regulator gene *aphA* [148,149].

*V. cholerae* employs QS systems to temporally control virulence during infection. It has been shown that QS represses virulence gene expression and biofilm formation while activating production of extracellular proteases, suggesting the importance of QS in entering and exiting the host [15,54,150,151]. Surface-attached *V. cholerae* (as in biofilms) may be the major entry route for *V. cholerae* infection as simple filtration using used sari cloths reduces cholera significantly [152]. The biofilm structure may be critical during entry into the host in order to protect against acid shock in the stomach [15]. After reaching the intestine, dispersal of individual cells from the biofilm leads *V. cholerae* to transition away from a high cell density state, leading to de-repression of virulence. However, the reduction in cell density alone may not be sufficient to completely inhibit HapR-mediated repression of virulence gene expression. By penetrating mucus barrier, *V. cholerae* utilize flagellar regulatory systems to further repress *hapR* [153]. Later in the infection, the number of *V. cholerae* in the intestine increases, and quorum sensing again represses CT and TCP production and activates production of proteases, which serve as detachases [154]. Detachment from the epithelium could permit individual cells to establish new infection foci in the intestine or to exit the host. This mucosal escape response is also mediated by the stationary phase alternative sigma factor RpoS [155]. In addition to these quorum sensing molecules, it has been shown that other small molecule metabolites, such as indole and cyclo(Phe-Pro) (cFP) that accumulate to high concentrations at the stationary phase, inhibit virulence through a global regulator LeuO [156,157]. The QS regulon also consists of a number of additional genes involved in chitin-induced natural competence, stress responses, and hemolysin production, phage production, among others [113,158–161], suggesting of the importance of QS mechanisms to adaptation to diverse environmental niches [136].

Interestingly, a large percentage of natural *V. cholerae* isolates are QS-deficient [162–164], implying a selective advantage of QS mutants in nature. It has been reported that these QS mutants actively cheat through signaling others to produce QS-dependent “public goods”[165], suggesting that social cheating may drive QS deficiency emergence within *V. cholerae* natural populations. The loss of QS regulation may also improve virulence gene expression in the gut.

## The role of the gut microbiome in *V. cholerae* pathogenesis

The gut microbiome is a highly diverse assemblage of microorganisms representing all domains of life, though dominated by the eubacteria. While many human body sites host resident or transiently present microbes, the gastrointestinal tract represents the densest site of continuous microbial colonization; it is thought that bacterial cells in the gut outnumber human somatic cells, and the genetic diversity of the assembled genomes of gut resident species far eclipses that of humans [166,167]. The gut microbiome has been implicated in numerous host phenotypes, and research has recently begun to focus on the molecular interactions of this commensal microbial community and enteric pathogens such as *V. cholerae* (Figure 2).

### Impact of cholera on the gut microbiome

The characteristic “rice water stool” produced during acute cholera is dominated by vibrios [168], and was an early clue as to the etiology of the disease in humans. Culture-dependent and culture-independent efforts have since defined at a much greater taxonomic resolution the effects of cholera on the bacteria within human gut microbiome. The fulminant diarrhea caused by cholera has predictably deleterious effects on the abundance and diversity of the gut microbial population. Culturing studies have shown a multi-log decrease in cultivatable non-*Vibrio* bacteria during cholera in stool during acute disease in adults compared to convalescent populations [168]. More recently, metagenomic techniques, focusing largely on high-throughput sequencing of PCR amplicons of the 16S ribosomal small subunit gene in fecal specimens, have been used to probe for gut microbiome changes at high taxonomic resolution. Hsiao et al. closely tracked adult cholera patients in Bangladesh from presentation at clinic to 3 months of convalescence, and showed that the microbiome became dramatically less diverse during diarrhea, becoming dominated by mostly streptococcal species [169]. Existing gut microbes were detected at very low abundance, but over the course of recovery from diarrhea expanded to re-populate the gut in a successional process similar to the initial colonization of the gut during infancy and childhood. This drop in diversity is paralleled in findings in children with cholera [170]. Subsequent 16S amplicon sequencing-based studies in malnourished children with cholera showed an increase in Enterobacteriaceae, Veillonellaceae, and Streptococcaceae during infection [171]. A transient but dramatic dysbiosis in microbial structure has since been reported not only for cholera, but diarrhea of multiple etiologies [172,173] and in severe malnutrition [174]. These patterns in fecal microbiome complexity and membership during dysbiosis are paralleled in the small intestine, where *E. coli*, Streptococci, and aerobic lactobacilli were found in abundance in the duodenum and jejunum by culturing [175].

### Gut microbiome structure as a driver of colonization resistance

A role for commensal microbes in *V. cholerae* infection outcome has been long recognized. Freter et al. showed in the 1950s that guinea pigs and mice whose commensal flora had been depleted by antibiotics were susceptible to *V. cholerae* colonization, while untreated animals were highly resistant [176]. This is in contrast to germfree mice, which support very high levels of *V. cholerae* colonization in both the distal small intestine and distal gut compartments [177]. Limitations in the ability to define, culture, and establish *in vivo* complex mixtures of microbes in the gut has until recently prevented work at the species resolution on the role of the human, as opposed to the rodent, gut microbiome, which differ in both species richness and composition [178].

Several recent studies have used a variety of molecular and animal model approaches to examine the role of human microbiome structure in susceptibility to infection. Hsiao et al. established in germfree mice defined communities of cultured human gut commensal bacteria closely modeled on normal healthy human gut communities [169]. The defined model healthy human microbiome were found to be highly resistant to *V. cholerae*, and one gut microbe commonly found in healthy human populations, *Blautia* (formerly *Ruminococcus*) *obeum* drove a large proportion of this colonization resistance phenotype. Specific exclusion of this species in communities established in germfree mice yielded significantly higher *V. cholerae* colonization, and direct competition of *V. cholerae* and *B. obeum* led to a 2-log decrease in pathogen load compared to germfree conditions. This suggested that human microbiomes could be a susceptibility factor for *V. cholerae* colonization, and that these effects may be highly specific to certain microbiome members; engineered communities with and without *B. obeum* in the above study had negligible differences in overall phylogenetic diversity, and the closely related *Blautia torques* showed no effects on *V. cholerae*. Midani et al. examined complete human fecal microbiomes of household contacts of cholera patients in Bangladesh that did or did not subsequently develop symptomatic infection to identify microbial correlates of infection susceptibility in full human microbiomes [179]. Using a machine learning approach, they identified several species associated with household contacts that remained uninfected, including *Blautia, Ruminococcus*, and *Prevotella* species, while *Streptococcus, Prevotella*, and *Blautia* species were higher in infected contacts. Differences in alpha diversity were not associated with subsequent infection. That species of the same genus were associated with both infected and uninfected outcomes further highlights the likely specificity of species and their biochemical functions in determining the outcome of exposure to *V. cholerae.*

Work by Alavi et al. has experimentally confirmed the role of differences in human gut microbiomes in *V. cholerae* colonization outcome [180]. These studies established defined model and complete human fecal microbiomes in germfree mice and suckling animals with their native murine microbiomes cleared using antibiotics. High-*Streptococcus* microbiomes modeled after diarrhea- and malnutrition-disrupted gut communities were highly susceptible to *V. cholerae* infection. Surprisingly, complete fecal microbiomes from healthy US donors transplanted into suckling animals yielded ~30-fold differences in subsequent *V. cholerae* colonization. These findings suggest that commensal microbe-dependent disease susceptibility differences are not strictly dichotomous between “normal” vs “diseased” microbiome states, and that interpersonal differences in microbiome structure can drive infection outcomes. Furthermore, this suggests that disruption of the microbiome by other infectious diarrheas or malnutrition may also be a risk factor for cholera.

### Commensal microbial quorum sensing in *V. cholerae* infection

The complex and tightly coordinated virulence regulatory cascade of *V. cholerae* can be modulated by autoinducer molecules from various sources. One mechanism of interaction between commensal microbes and *V. cholerae* pathogenesis is the production of cross-species autoinducers. *V. cholerae* responds to a set of species-specific (CAI-1) and potentially cross-species (AI-2, DPO, ethanolamine) signals in gene regulation [144,149,181]. The human commensal *B. obeum* has been shown to upregulate production of an AI-2 molecule in response to *V. cholerae* infection when colonized in germfree mice [169]. Expression of the *B. obeum* AI-2 synthase LuxS in an AI-2-null *E. coli* was sufficient to restrict *V. cholerae* colonization. This signaling was independent of the canonical AI-2 sensor LuxP, as *luxP* deletion did not rescue *V. cholerae* colonization in the presence of *B. obeum*. This suggests that AI-2 molecules produced by different members of the gut microbiome may differentially integrate into the virulence regulatory cascade. LuxS homologs are widely distributed in the genomes of gut commensals [182], though it is uncertain if there is substantial structural diversity in the resulting autoinducer molecules. Two structures of AI-2 have been elucidated, the AI-2 of *Vibrio* being a furanosyl borate diester, in contrast to a non-borated molecule synthesized by *Salmonella* [140,183]. Cross-feeding experiments demonstrate that *V. cholerae* can respond to AI-2 from *E. coli* [184]. That LuxP seems to be dispensable in *V. cholerae* regulatory responses to *B. obeum* AI-2 suggests that there may be further structural diversity in this QS molecule as made by different gut commensals.

### Commensal microbial bile metabolism modulates V. cholerae virulence

Similarly to *V. cholerae*, commensal gut microbes adapted to the small intestine have evolved mechanisms to deal with the bacteriostatic qualities of bile. Microbial bile salt hydrolase (*bsh*) enzymes can remove the conjugated amino acid of primary bile salts, and a series of microbial enzymes can dehydroxylate the 7^th^ position of sterol backbone of bile (7α-dehydroxylation). Microbial *bsh* enzymes are broadly distributed within the genomes of human gut-associated species and play an important role in microbial bile tolerance and bile recirculation. Deconjugation of amino acids attached to primary bile salts such as taurocholate and glycocholate reduces the hydrophilicity of these molecules, leading to a drop in their detergent-like effects [185,186]. Though many gut commensal microbes are refractory to genetic manipulation, mutations in *bsh* genes in *Lactobacillus amylovorus* and *L. plantarum* have been associated with sensitivity to bile and bile salts [186,187]. That microbial metabolism of bile salts is a major player in the composition of the bile acid pool has been demonstrated in germfree animal systems; in germfree mice, essentially all small intestinal bile are conjugated primary bile salts (taurocholate and tauromuricholate) [188]. Bioinformatic studies have identified several broad *bsh* phylotypes within commensal microorganisms, which have different predicted bile salt substrates and activity [58]. The presence of specific sets of bacteria in the small intestine can thus lead to differential activity against components of the bile acid pool, with consequent effects on the chemical environment of the small bowel.

Recent work has shown that *bsh* activity by *V. cholerae*-restricting microbes such as *B. obeum* strongly contributes to the outcome of *V. cholerae* infection [180]. Using an *in vitro* screen for activity against bile acids, Alavi et al. showed that *B. obeum* encodes for a *bsh* level with high activity against taurocholate, a key activator of *V. cholerae* virulence in mice and humans [68]. Overall levels of *bsh* activity were higher in organisms characteristic of healthy human gut microbiomes *in vitro* and *bsh* genes were more abundant in sequenced metagenomic datasets of fecal samples from healthy Bangladeshi adults compared to dysbiotic, *V. cholerae*-susceptible microbiomes. Genes encoding for these enzymes were found in lower abundance in post-diarrhea fecal microbiomes, and taurocholate deconjugating activity was less frequently found in isolates of species characteristic of these dysbiotic gut communities. *B. obeum bsh* activity has been directly correlated to the ability of complete complex human fecal microbiomes to resist *V. cholerae* infection in suckling mice; communities with *B. obeum* led to lower *tcpA* expression during infection of suckling animals [180]. This was independent of AI-2 production, as *B. obeum* reduced *tcpA* activation by intestinal tissues even when boiled to remove AI-2, and constitutive expression of *B. obeum bsh* was able to significantly restrict *V. cholerae* colonization. This suggests that microbiome dysbiosis can lead to increased *V. cholerae* susceptibility via a loss of *bsh* activity, and represents a recurrent and individual-specific window of vulnerability to infection in human populations in cholera endemic areas.

### Driving disease resistance using the microbiome

Several proof-of-principle studies have demonstrated the effectiveness of engineering probiotic strains to modulate *V. cholerae* colonization *in vivo*. Duan et al. used a probiotic *E. coli* Nissle strain engineered to produce CAI-1 via constitutive *cqsA* expression, and showed that introduction of this strain into suckling mice reduced susceptibility to subsequent *V. cholerae* challenge in a QS-dependent manner [189]. Recently, Mao et al. reported the effectiveness of an engineered *Lactococcus lactis* that can serve as a sensor of *V. cholerae* infection via detection of *V. cholerae*-specific autoinducers and consequent production of an enzymatic indicator. This strain was also able to promote colonization resistance via decreases in intestinal pH [190]. Co-opting the metabolic activities of commensal microbes has also been shown to reduce *V. cholerae* susceptibility. Expression of other autoinducers produced by microbiome members such as AI-2 (via *luxS* expression) [169] and manipulation of bile acid pools (via *bsh* expression) [180] has demonstrated effectiveness in mouse infection models, using *E. coli* as an expression platform. Engineered *V. cholerae* may also have “probiotic” effects on subsequent infection; recent studies have shown that a non-virulent candidate live vaccine strain rapidly outcompeted fully virulent *V. cholerae* in infant rabbits [191]. However, it is unclear whether the use of existing probiotic species for introduction of specific microbial functions will have durable efficacy as a prophylactic compared to introduction of fully gut-adapted species with multiple levels of activity; commercial probiotic strains have demonstrated low effective colonization in germfree animals [192] and *V. cholerae* are rapidly excluded by normal commensal microbes in animal models in the absence of successful colonization leading to toxin production and diarrhea [169]. The identification of human commensal microbes with efficacy against *V. cholerae* virulence, for example *B. obeum*, the source organism for the *luxS* and *bsh* mechanisms described above, may allow for more stable prophylactic effects on the host due to these organisms being better adapted to colonization of the human gastrointestinal tract.

### Inter-bacterial competition and V. cholerae virulence

*V. cholerae* has evolved numerous mechanisms as part of infection that are able to deplete the native microflora of the small intestine. The profuse watery diarrhea of cholera is one obvious element of this process, as this rapidly drops the levels of commensal microbes in both rice water stool and the small intestine [168,169,175]. Recent work has identified other biochemical pathways by which this human pathogen can influence gut commensal levels. The Type-six secretion system (T6SS) is a molecular complex that enables *V. cholerae* to kill other Gram-negative cells and modulate host cell behavior via contact-dependent cell-cell secretion of effector proteins [193,194]. These systems have been identified in several Gram-negative species associated with the GI tract [195,196]. Several lines of evidence suggest that this mechanism of microbial competition plays an important role in *V. cholerae* pathogenesis. Genetic deletion of the T6SS leads to a colonization defect or loss of virulence in several animal models, including infant rabbits [197], *Drosophila* [198] and suckling mice [180,199]. *V. cholerae* uses the T6SS to directly and indirectly target competitor commensal microbes. This interbacterial competition can be via direct killing; *V. cholerae* is able to use T6SS to kill murine commensal *E. coli* isolates during colonization of suckling mice [199]. Interestingly, this killing leads to an increased upregulation of virulence gene expression over and above that seen during the *in vitro* to *in vivo* transition. This has also been demonstrated in a *Drosophila* model of infection, where T6SS interactions with the commensal *Acetobacter pasteurianus* led to increased disease symptoms including host death [198]. T6SS can also play a role in displacing competitive commensal organisms as demonstrated in *V. cholerae* colonization of zebrafish [200], where T6SS mediated increased gut motility to provide a competitive advantage to the pathogen over resident bacteria.

The T6SS may also be regulated by processes related to commensal metabolism in the gut. Several studies have also shown a link between QS and T6SS regulation; the key QS regulator HapR has been shown to directly initiate T6SS gene expression [201] and indirectly via the transcription factor QstR [202]. QS-depending T6SS regulation has been shown to be conserved across pandemic and non-pandemic strains of *V. cholerae* [203]. The presence of numerous inter-species signaling molecules in the gut may thus also be associated with upregulation of competitive cell-cell killing mechanisms during infection. The layer of highly glycosylated mucins that form the intestinal epithelial mucus barrier is also sensed by *V. cholerae* to de-repress expression of T6SS related genes [204]. T6SS gene expression can also be regulated by specific bile acids; deoxycholic acid, a metabolic product of cholic acid formed by 7-α-dehydroxylation by microbial action, has been shown to inhibit T6SS assembly [204]. These processes may intersect with the action of the gut microbiome. Commensal microbes are found in large numbers in mucus [205,206]; several microbes, for example *Akkermansia muciniphila* [207], are known to be highly adapted to use human mucus components as nutritional sources, while the role of microbial enzymes in bile metabolism has been heavily studied. Processing by commensal microorganisms of mucin and bile may thus play important but as yet un-elucidated roles in T6SS regulation.

## Perspectives and future studies

As experimental and bioinformatic techniques for probing microbe-microbe and microbe-host interactions during *V. cholerae* infections advance, we can expect new discoveries in how the gut microbiome shapes the *in vivo* signals that drive *V. cholerae* infection.

While oral rehydration with antibiotic treatment is highly effective at reducing mortality due to cholera, cholera remains a formidable global health challenge demanding of new therapeutic or prophylactic approaches. While the development of antibiotic resistance in *V. cholerae* is of growing concern [208], therapeutic or prophylactic approaches using the microbiome that target colonization, virulence gene expression, or nutrient competition is unlikely to be as strong a driver for the development of resistance mechanisms. Therapies leading to reductions in mortality also largely do not reduce the high morbidity of cholera. It is increasingly clear that the variability of the microbiome, from individual to individual, and within individuals in response to environmental stimuli, determines a personalized infection outcome, depending on the presence and activity of key commensal microbes. Thus, manipulation of microbial colonization, if understood mechanistically and rationally designed, may offer durable prophylactic or therapeutic efficacy against cholera. Probiotics that can durably establish in the context of complex human fecal gut communities, as opposed to simplified animal model system, critical for such approaches; the identification of commensals such as *B. obeum* with anti-virulence activity that are native to the human gastrointestinal tract and able to establish colonization across different human gut microbiomes is thus of increasing significance [169,180].

Another avenue of research of key importance to the field is the development of new and tractable experimental models to identify candidate microbes mediating infection resistance and to test targeted microbiome manipulations. Mice without native murine microbes, whether germfree [169,180,192] or antibiotic-cleared [199], can serve as hosts for complete or defined model human fecal gut microbiomes in the context of *V. cholerae* colonization and infection. These systems allow researchers to insert, remove, or mix microbes and assemblages to determine their effects on pathogenesis and *in vivo* pathogen fitness. While *V. cholerae* behavior differs in germfree adult mice compared to suckling animals by lower CT expression [169], this experimental model allowed for the targeted establishment of human gut bacteria to evaluate their effects on colonization, and extended studies of *in vivo* metabolism and cell–cell interactions that are difficult to accomplish with shorter-term models such as suckling mice or suckling rabbits.

Consistency of effects on colonization resistance will be of key importance in identifying candidate next-generation probiotics. Combinatorial approaches, where microbial subsets are randomized to determine whether specific microbes play active roles *in vivo* regardless of other colonizing microbes, have also been used to identify contributors to host immunity and *V. cholerae* colonization resistance in the context of quite different background microbiomes [180,209], a necessary characteristic in a broadly efficacious next-generation probiotic.

The role of diet and nutrition, both of the host and of *V. cholerae* during colonization, may also play an important role in future work. Microbiome structure is strongly driven by host diet [210–213]. However, the role of dietary components, and what nutrient sources are most important for rapid *V. cholerae* proliferation *in vivo*, have not been extensively studied. The role of commensal microbes in setting the stage for early infection through contribution to the gut nutrient landscape is also largely unknown. Prebiotic approaches, which use targeted nutritional interventions to drive specific effects on gut microbiomes, may also be a fruitful area of research for targeted microbiome modification as applied to *V. cholerae* colonization resistance.

The expansion in multi-omics approaches, microbial ecological analysis, and experimental animal models of *V. cholerae* infection and *in vivo* fitness have revealed numerous fascinating aspects of the biology of this important human pathogen and how it is impacted by the variable membership and biochemistry of the human gut microbiome. Future advances will capitalize on the translational potential of these findings, as pathogenesis starts to incorporate new understandings of the microbiome to produce prophylactic and therapeutic interventions for cholera.

## References

[1] (WHO) WHO. Cholera annual report 2017. Weekly Epidemiological Rec. 2018;93:489–500.

[2] Faruque SM, Albert MJ, Mekalanos JJ. Epidemiology, genetics, and ecology of toxigenic Vibrio cholerae. Microbiol Mol Biol Rev. 1998;62:1301–1314. 9841673 10.1128/mmbr.62.4.1301-1314.1998 PMC98947

[3] Sack DA, Sack RB, Nair GB, et al. Cholera. Lancet. 2004;363:223–233. 14738797 10.1016/s0140-6736(03)15328-7

[4] Clemens JD, Nair GB, Ahmed T, et al. Cholera. Lancet. 2017;390:1539–1549. 28302312 10.1016/S0140-6736(17)30559-7

[5] Hsueh BY, Waters CM. Combating cholera. F1000Res. 2019;8:589. 31069064 10.12688/f1000research.18093.1 PMC6492228

[6] Reduction in cholera deaths targeted for 2030. Nat Microbiol. 2017;2:1457. 29070822 10.1038/s41564-017-0055-9

[7] Kaper JB, Morris JG Jr., Levine MM. Cholera. Clin Microbiol Rev. 1995;8:48–86. 7704895 10.1128/cmr.8.1.48 PMC172849

[8] Watnick PI, Kolter R. Steps in the development of a Vibrio cholerae El Tor biofilm. Mol Microbiol. 1999;34:586–595. 10564499 10.1046/j.1365-2958.1999.01624.x PMC2860543

[9] Thelin KH, Taylor RK. Toxin-coregulated pilus, but not mannose-sensitive hemagglutinin, is required for colonization by Vibrio cholerae O1 El Tor biotype and O139 strains. Infect Immun. 1996;64:2853–2856. 8698524 10.1128/iai.64.7.2853-2856.1996 PMC174155

[10] Colwell RR, Huq A. Environmental reservoir of Vibrio cholerae. The causative agent of cholera. Ann N Y Acad Sci. 1994;740:44–54. 7840478 10.1111/j.1749-6632.1994.tb19852.x

[11] Colwell RR, Kaper J, Joseph SW. Vibrio cholerae, Vibrio parahaemolyticus, and other vibrios: occurrence and distribution in Chesapeake Bay. Science. 1977;198:394–396. 910135

[12] Huq A, Small EB, West PA, et al. Ecological relationships between Vibrio cholerae and planktonic crustacean copepods. Appl Environ Microbiol. 1983;45:275–283. 6337551 10.1128/aem.45.1.275-283.1983 PMC242265

[13] Tamplin ML, Gauzens AL, Huq A, et al. Attachment of Vibrio cholerae serogroup O1 to zooplankton and phytoplankton of Bangladesh waters. Appl Environ Microbiol. 1990;56:1977–1980. 2383016 10.1128/aem.56.6.1977-1980.1990 PMC184543

[14] Colwell RR, Brayton PR, Grimes DJ, et al. Viable but non-culturable vibrio cholerae and related pathogens in the environment: implications for release of genetically engineered microorganisms. Bio/Technology. 1985;3:817–820.

[15] Zhu J, Mekalanos JJ. Quorum sensing-dependent biofilms enhance colonization in Vibrio cholerae. Dev Cell. 2003;5:647–656. 14536065 10.1016/s1534-5807(03)00295-8

[16] Hay AJ, Zhu J. Host intestinal signal-promoted biofilm dispersal induces Vibrio cholerae colonization. Infect Immun. 2015;83:317–323. 25368110 10.1128/IAI.02617-14 PMC4288906

[17] Waldor MK, Mekalanos JJ. Lysogenic conversion by a filamentous phage encoding cholera toxin. Science. 1996;272:1910–1914. 8658163 10.1126/science.272.5270.1910

[18] Miller VL, Taylor RK, Mekalanos JJ. Cholera toxin transcriptional activator toxR is a transmembrane DNA binding protein. Cell. 1987;48:271–279. 3802195 10.1016/0092-8674(87)90430-2

[19] Herrington DA, Hall RH, Losonsky G, et al. Toxin, toxin-coregulated pili, and the toxR regulon are essential for Vibrio cholerae pathogenesis in humans. J Exp Med. 1988;168:1487–1492. 2902187 10.1084/jem.168.4.1487 PMC2189073

[20] Shaw CE, Taylor RK. Vibrio cholerae O395 tcpA pilin gene sequence and comparison of predicted protein structural features to those of type 4 pilins. Infect Immun. 1990;58:3042–3049. 1974887 10.1128/iai.58.9.3042-3049.1990 PMC313608

[21] Karaolis DK, Johnson JA, Bailey CC, et al. A Vibrio cholerae pathogenicity island associated with epidemic and pandemic strains. Proc Natl Acad Sci U S A. 1998;95:3134–3139. 9501228 10.1073/pnas.95.6.3134 PMC19707

[22] DiRita VJ. Co-ordinate expression of virulence genes by ToxR in Vibrio cholerae. Mol Microbiol. 1992;6:451–458. 1560773 10.1111/j.1365-2958.1992.tb01489.x

[23] DiRita VJ, Mekalanos JJ. Periplasmic interaction between two membrane regulatory proteins, ToxR and ToxS, results in signal transduction and transcriptional activation. Cell. 1991;64:29–37. 1898871 10.1016/0092-8674(91)90206-e

[24] Gallegos MT, Schleif R, Bairoch A, et al. Arac/XylS family of transcriptional regulators. Microbiol Mol Biol Rev. 1997;61:393–410. 9409145 10.1128/mmbr.61.4.393-410.1997 PMC232617

[25] Lowden MJ, Skorupski K, Pellegrini M, et al. Structure of Vibrio cholerae ToxT reveals a mechanism for fatty acid regulation of virulence genes. Proc Natl Acad Sci U S A. 2010;107:2860–2865. 20133655 10.1073/pnas.0915021107 PMC2840316

[26] Cruite JT, Kovacikova G, Clark KA, et al. Structural basis for virulence regulation in Vibrio cholerae by unsaturated fatty acid components of bile. Commun Biol. 2019;2:440. 31815195 10.1038/s42003-019-0686-x PMC6882843

[27] Schuhmacher DA, Klose KE. Environmental signals modulate ToxT-dependent virulence factor expression in Vibrio cholerae. J Bacteriol. 1999;181:1508–1514. 10049382 10.1128/jb.181.5.1508-1514.1999 PMC93540

[28] Matson JS, Withey JH, DiRita VJ. Regulatory networks controlling Vibrio cholerae virulence gene expression. Infect Immun. 2007;75:5542–5549. 17875629 10.1128/IAI.01094-07 PMC2168339

[29] Kovacikova G, Lin W, Taylor RK, et al. The fatty acid regulator FadR influences the expression of the virulence cascade in the el tor biotype of vibrio cholerae by modulating the Levels of ToxT via two different mechanisms. J Bacteriol. 2017;199:e00762-16. 10.1128/JB.00762-16 PMC5350278 28115548

[30] Skorupski K, Taylor RK. Control of the ToxR virulence regulon in Vibrio cholerae by environmental stimuli. Mol Microbiol. 1997;25:1003–1009. 9350858 10.1046/j.1365-2958.1997.5481909.x

[31] Higgins DE, DiRita VJ. Transcriptional control of toxT, a regulatory gene in the ToxR regulon of Vibrio cholerae. Mol Microbiol. 1994;14:17–29. 7830555 10.1111/j.1365-2958.1994.tb01263.x

[32] Hase CC, Mekalanos JJ. TcpP protein is a positive regulator of virulence gene expression in Vibrio cholerae. Proc Natl Acad Sci U S A. 1998;95:730–734. 9435261 10.1073/pnas.95.2.730 PMC18489

[33] Childers BM, Klose KE. Regulation of virulence in Vibrio cholerae: the ToxR regulon. Future Microbiol. 2007;2:335–344. 17661707 10.2217/17460913.2.3.335

[34] Bina J, Zhu J, Dziejman M, et al. ToxR regulon of Vibrio cholerae and its expression in vibrios shed by cholera patients. Proc Natl Acad Sci U S A. 2003;100:2801–2806. 12601157 10.1073/pnas.2628026100 PMC151421

[35] Miller VL, Mekalanos JJ. A novel suicide vector and its use in construction of insertion mutations - osmoregulation of outer-membrane proteins and virulence determinants in vibrio-cholerae requires toxr. J Bacteriol. 1988;170:2575–2583. 2836362 10.1128/jb.170.6.2575-2583.1988 PMC211174

[36] Provenzano D, Lauriano CM, Klose KE. Characterization of the role of the ToxR-modulated outer membrane porins OmpU and OmpT in Vibrio cholerae virulence. J Bacteriol. 2001;183:3652–3662. 11371530 10.1128/JB.183.12.3652-3662.2001 PMC95243

[37] Miller VL, DiRita VJ, Mekalanos JJ. Identification of toxS, a regulatory gene whose product enhances toxR-mediated activation of the cholera toxin promoter. J Bacteriol. 1989;171:1288–1293. 2646275 10.1128/jb.171.3.1288-1293.1989 PMC209743

[38] Almagro-Moreno S, Root MZ, Taylor RK. Role of ToxS in the proteolytic cascade of virulence regulator ToxR in Vibrio cholerae. Mol Microbiol. 2015;98:963–976. 26316386 10.1111/mmi.13170

[39] Almagro-Moreno S, Kim TK, Skorupski K, et al. Proteolysis of virulence regulator ToxR is associated with entry of Vibrio cholerae into a dormant state. PLoS Genet. 2015;11:e1005145. 25849031 10.1371/journal.pgen.1005145 PMC4388833

[40] Lembke M, Hofler T, Walter AN, et al. Host stimuli and operator binding sites controlling protein interactions between virulence master regulator ToxR and ToxS in Vibrio cholerae. Mol Microbiol. 2020;114:262–278 10.1111/mmi.14510 PMC7496328 32251547

[41] Beck NA, Krukonis ES, DiRita VJ. TcpH influences virulence gene expression in Vibrio cholerae by inhibiting degradation of the transcription activator TcpP. J Bacteriol. 2004;186:8309–8316. 15576780 10.1128/JB.186.24.8309-8316.2004 PMC532408

[42] Matson JS, DiRita VJ. Degradation of the membrane-localized virulence activator TcpP by the YaeL protease in Vibrio cholerae. Proc Natl Acad Sci U S A. 2005;102:16403–16408. 16254052 10.1073/pnas.0505818102 PMC1283431

[43] Morgan SJ, French EL, Thomson JJ, et al. Formation of an intramolecular periplasmic disulfide bond in TcpP protects TcpP and TcpH from degradation in vibrio cholerae. J Bacteriol. 2016;198:498–509. 26574510 10.1128/JB.00338-15 PMC4719457

[44] Goss TJ, Seaborn CP, Gray MD, et al. Identification of the TcpP-binding site in the toxT promoter of Vibrio cholerae and the role of ToxR in TcpP-mediated activation. Infect Immun. 2010;78:4122–4133. 20679441 10.1128/IAI.00566-10 PMC2950353

[45] Krukonis ES, Yu RR, Dirita VJ. The Vibrio cholerae ToxR/TcpP/ToxT virulence cascade: distinct roles for two membrane-localized transcriptional activators on a single promoter. Mol Microbiol. 2000;38:67–84. 11029691 10.1046/j.1365-2958.2000.02111.x

[46] Murley YM, Carroll PA, Skorupski K, et al. Differential transcription of the tcpPH operon confers biotype-specific control of the Vibrio cholerae ToxR virulence regulon. Infect Immun. 1999;67:5117–5123. 10496885 10.1128/iai.67.10.5117-5123.1999 PMC96860

[47] Goss TJ, Morgan SJ, French EL, et al. ToxR recognizes a direct repeat element in the toxT, ompU, ompT, and ctxA promoters of Vibrio cholerae to regulate transcription. Infect Immun. 2013;81:884–895. 23297386 10.1128/IAI.00889-12 PMC3584884

[48] Krukonis ES, DiRita VJ. DNA binding and ToxR responsiveness by the wing domain of TcpP, an activator of virulence gene expression in Vibrio cholerae. Mol Cell. 2003;12:157–165. 12887901 10.1016/s1097-2765(03)00222-3

[49] De Silva RS, Kovacikova G, Lin W, et al. Crystal structure of the virulence gene activator AphA from Vibrio cholerae reveals it is a novel member of the winged helix transcription factor superfamily. J Biol Chem. 2005;280:13779–13783. 15647287 10.1074/jbc.M413781200 PMC2652724

[50] Kovacikova G, Lin W, Skorupski K. Vibrio cholerae AphA uses a novel mechanism for virulence gene activation that involves interaction with the LysR-type regulator AphB at the tcpPH promoter. Mol Microbiol. 2004;53:129–142. 15225309 10.1111/j.1365-2958.2004.04121.x

[51] Xu X, Stern AM, Liu Z, et al. Virulence regulator AphB enhances toxR transcription in Vibrio cholerae. BMC Microbiol. 2010;10:3. 20053280 10.1186/1471-2180-10-3 PMC2806343

[52] Kovacikova G, Skorupski K. Regulation of virulence gene expression in Vibrio cholerae by quorum sensing: hapR functions at the aphA promoter. Mol Microbiol. 2002;46:1135–1147. 12421317 10.1046/j.1365-2958.2002.03229.x

[53] Lin W, Kovacikova G, Skorupski K. The quorum sensing regulator HapR downregulates the expression of the virulence gene transcription factor AphA in Vibrio cholerae by antagonizing Lrp- and VpsR-mediated activation. Mol Microbiol. 2007;64:953–967. 17501920 10.1111/j.1365-2958.2007.05693.x

[54] Zhu J, Miller MB, Vance RE, et al. Quorum-sensing regulators control virulence gene expression in Vibrio cholerae. Proc Natl Acad Sci U S A. 2002;99:3129–3134. 11854465 10.1073/pnas.052694299 PMC122484

[55] Hofmann AF. Bile acids: the good, the bad, and the ugly. News Physiol Sci. 1999;14:24–29. 11390813 10.1152/physiologyonline.1999.14.1.24

[56] Ridlon JM, Kang DJ, Hylemon PB. Bile salt biotransformations by human intestinal bacteria. J Lipid Res. 2006;47:241–259. 16299351 10.1194/jlr.R500013-JLR200

[57] Jones BV, Begley M, Hill C, et al. Functional and comparative metagenomic analysis of bile salt hydrolase activity in the human gut microbiome. Proc Natl Acad Sci U S A. 2008;105:13580–13585. 18757757 10.1073/pnas.0804437105 PMC2533232

[58] Song Z, Cai Y, Lao X, et al. Taxonomic profiling and populational patterns of bacterial bile salt hydrolase (BSH) genes based on worldwide human gut microbiome. Microbiome. 2019;7:9. 30674356 10.1186/s40168-019-0628-3 PMC6345003

[59] Dawson PA, Karpen SJ. Intestinal transport and metabolism of bile acids. J Lipid Res. 2015;56:1085–1099. 25210150 10.1194/jlr.R054114 PMC4442867

[60] Di Ciaula A, Garruti G, Lunardi Baccetto R, et al. Bile Acid Physiology. Ann Hepatol. 2017;16(Suppl 1):S4–S14. 10.5604/01.3001.0010.5493 29080336

[61] Begley M, Gahan CG, Hill C. The interaction between bacteria and bile. FEMS Microbiol Rev. 2005;29:625–651. 16102595 10.1016/j.femsre.2004.09.003

[62] Bina XR, Provenzano D, Nguyen N, et al. Vibrio cholerae RND family efflux systems are required for antimicrobial resistance, optimal virulence factor production, and colonization of the infant mouse small intestine. Infect Immun. 2008;76:3595–3605. 18490456 10.1128/IAI.01620-07 PMC2493215

[63] Simonet VC, Basle A, Klose KE, et al. The Vibrio cholerae porins OmpU and OmpT have distinct channel properties. J Biol Chem. 2003;278:17539–17545. 12606562 10.1074/jbc.M301202200

[64] Provenzano D, Klose KE. Altered expression of the ToxR-regulated porins OmpU and OmpT diminishes Vibrio cholerae bile resistance, virulence factor expression, and intestinal colonization. Proc Natl Acad Sci U S A. 2000;97:10220–10224. 10944196 10.1073/pnas.170219997 PMC27820

[65] Cerda-Maira FA, Ringelberg CS, Taylor RK. The bile response repressor BreR regulates expression of the Vibrio cholerae breAB efflux system operon. J Bacteriol. 2008;190:7441–7452. 18776020 10.1128/JB.00584-08 PMC2576662

[66] Butler SM, Camilli A. Going against the grain: chemotaxis and infection in Vibrio cholerae. Nat Rev Microbiol. 2005;3:611–620. 16012515 10.1038/nrmicro1207 PMC2799996

[67] Gupta S, Chowdhury R. Bile affects production of virulence factors and motility of Vibrio cholerae. Infect Immun. 1997;65:1131–1134. 9038330 10.1128/iai.65.3.1131-1134.1997 PMC175102

[68] Yang M, Liu Z, Hughes C, et al. Bile salt-induced intermolecular disulfide bond formation activates Vibrio cholerae virulence. Proc Natl Acad Sci U S A. 2013;110:2348–2353. 23341592 10.1073/pnas.1218039110 PMC3568309

[69] Xue Y, Tu F, Shi M, et al. Redox pathway sensing bile salts activates virulence gene expression in Vibrio cholerae. Mol Microbiol. 2016;102:909–924. 27610607 10.1111/mmi.13497

[70] Depuydt M, Messens J, Collet JF. How proteins form disulfide bonds. Antioxid Redox Signal. 2011;15:49–66. 20849374 10.1089/ars.2010.3575

[71] Hay AJ, Yang M, Xia X, et al. Calcium enhances bile salt-dependent virulence activation in vibrio cholerae. Infect Immun. 2017;85:e00707-16. 10.1128/IAI.00707-16 PMC5203667 27849180

[72] Lembke M, Pennetzdorfer N, Tutz S, et al. Proteolysis of ToxR is controlled by cysteine-thiol redox state and bile salts in Vibrio cholerae. Mol Microbiol. 2018;110:796–810. 30218472 10.1111/mmi.14125 PMC6242745

[73] Chatterjee A, Dutta PK, Chowdhury R. Effect of fatty acids and cholesterol present in bile on expression of virulence factors and motility of Vibrio cholerae. Infect Immun. 2007;75:1946–1953. 17261615 10.1128/IAI.01435-06 PMC1865667

[74] Wilson M. Microbial inhabitants of humans: their ecology and role in health and disease. Cambridge, England: Cambridge University Press; 2004.

[75] Marrero K, Sanchez A, Rodriguez-Ulloa A, et al. Anaerobic growth promotes synthesis of colonization factors encoded at the Vibrio pathogenicity island in Vibrio cholerae El Tor. Res Microbiol. 2009;160:48–56. 19015025 10.1016/j.resmic.2008.10.005

[76] Xu Q, Dziejman M, Mekalanos JJ. Determination of the transcriptome of Vibrio cholerae during intraintestinal growth and midexponential phase in vitro. Proc Natl Acad Sci U S A. 2003;100:1286–1291. 12552086 10.1073/pnas.0337479100 PMC298765

[77] Lee KM, Park Y, Bari W, et al. Activation of cholera toxin production by anaerobic respiration of trimethylamine N-oxide in Vibrio cholerae. J Biol Chem. 2012;287:39742–39752. 23019319 10.1074/jbc.M112.394932 PMC3501055

[78] Marteyn B, West NP, Browning DF, et al. Modulation of Shigella virulence in response to available oxygen in vivo. Nature. 2010;465:355–358. 20436458 10.1038/nature08970 PMC3750455

[79] Leclerc GJ, Tartera C, Metcalf ES. Environmental regulation of Salmonella typhi invasion-defective mutants. Infect Immun. 1998;66:682–691. 9453627 10.1128/iai.66.2.682-691.1998 PMC107957

[80] Khullar M, Singh RD, Smriti M, et al. Anaerobiosis-induced virulence of Salmonella enterica subsp. enterica serovar Typhimurium: role of phospholipase Cgamma signalling cascade. J Med Microbiol. 2003;52:741–745. 12909648 10.1099/jmm.0.05186-0

[81] Schuller S, Phillips AD. Microaerobic conditions enhance type III secretion and adherence of enterohaemorrhagic Escherichia coli to polarized human intestinal epithelial cells. Environ Microbiol. 2010;12:2426–2435. 10.1111/j.1462-2920.2010.02216.x PMC4966633 20406285

[82] Kovacikova G, Lin W, Skorupski K. The LysR-type virulence activator AphB regulates the expression of genes in Vibrio cholerae in response to low pH and anaerobiosis. J Bacteriol. 2010;192:4181–4191. 20562308 10.1128/JB.00193-10 PMC2916415

[83] Liu Z, Yang M, Peterfreund GL, et al. Vibrio cholerae anaerobic induction of virulence gene expression is controlled by thiol-based switches of virulence regulator AphB. Proc Natl Acad Sci U S A. 2011;108:810–815. 21187377 10.1073/pnas.1014640108 PMC3021084

[84] Liu Z, Wang H, Zhou Z, et al. Differential thiol-based switches jump-start vibrio cholerae pathogenesis. Cell Rep. 2016;14:347–354. 26748713 10.1016/j.celrep.2015.12.038 PMC4715633

[85] Fan F, Liu Z, Jabeen N, et al. Enhanced interaction of Vibrio cholerae virulence regulators TcpP and ToxR under oxygen-limiting conditions. Infect Immun. 2014;82:1676–1682. 24491579 10.1128/IAI.01377-13 PMC3993381

[86] Sengupta N, Paul K, Chowdhury R. The global regulator ArcA modulates expression of virulence factors in Vibrio cholerae. Infect Immun. 2003;71:5583–5589. 14500477 10.1128/IAI.71.10.5583-5589.2003 PMC201065

[87] Hengge R, Grundling A, Jenal U, et al. Bacterial signal transduction by Cyclic Di-GMP and other nucleotide second messengers. J Bacteriol. 2016;198:15–26. 26055111 10.1128/JB.00331-15 PMC4686208

[88] Skorupski K, Taylor RK. Cyclic AMP and its receptor protein negatively regulate the coordinate expression of cholera toxin and toxin-coregulated pilus in Vibrio cholerae. Proc Natl Acad Sci U S A. 1997;94:265–270. 8990197 10.1073/pnas.94.1.265 PMC19310

[89] Kovacikova G, Skorupski K. Overlapping binding sites for the virulence gene regulators AphA, AphB and cAMP-CRP at the Vibrio cholerae tcpPH promoter. Mol Microbiol. 2001;41:393–407. 11489126 10.1046/j.1365-2958.2001.02518.x

[90] Manneh-Roussel J, Haycocks JRJ, Magan A, et al. cAMP receptor protein controls vibrio cholerae gene expression in response to host colonization. mBio. 2018;9:e00966-18. 10.1128/mBio.00966-18 PMC6050953 29991587

[91] Hauryliuk V, Atkinson GC, Murakami KS, et al. Recent functional insights into the role of (p)ppGpp in bacterial physiology. Nature Rev Microbiol. 2015;13:298–309. 25853779 10.1038/nrmicro3448 PMC4659695

[92] Kim HY, Go J, Lee KM, et al. Guanosine tetra- and pentaphosphate increase antibiotic tolerance by reducing reactive oxygen species production in Vibrio cholerae. J Biol Chem. 2018;293:5679–5694. 29475943 10.1074/jbc.RA117.000383 PMC5900777

[93] Romling U, Galperin MY, Gomelsky M. Cyclic di-GMP: the first 25 years of a universal bacterial second messenger. Microbiol Mol Biol Rev. 2013;77:1–52. 23471616 10.1128/MMBR.00043-12 PMC3591986

[94] Conner JG, Zamorano-Sanchez D, Park JH, et al. The ins and outs of cyclic di-GMP signaling in Vibrio cholerae. Curr Opin Microbiol. 2017;36:20–29. 28171809 10.1016/j.mib.2017.01.002 PMC5534393

[95] Wu DC, Zamorano-Sanchez D, Pagliai FA, et al. Reciprocal c-di-GMP signaling: incomplete flagellum biogenesis triggers c-di-GMP signaling pathways that promote biofilm formation. PLoS Genet. 2020;16:e1008703. 32176702 10.1371/journal.pgen.1008703 PMC7098655

[96] Zamorano-Sanchez D, Xian W, Lee CK, et al. Functional specialization in vibrio cholerae diguanylate cyclases: distinct modes of motility suppression and c-di-GMP production. mBio. 2019;10:e00670-19. 10.1128/mBio.00670-19 PMC6479008 31015332

[97] Krasteva PV, Fong JC, Shikuma NJ, et al. Vibrio cholerae VpsT regulates matrix production and motility by directly sensing cyclic di-GMP. Science. 2010;327:866–868. 20150502 10.1126/science.1181185 PMC2828054

[98] Floyd KA, Lee CK, Xian W, et al. c-di-GMP modulates type IV MSHA pilus retraction and surface attachment in Vibrio cholerae. Nat Commun. 2020;11:1549. 32214098 10.1038/s41467-020-15331-8 PMC7096442

[99] Srivastava D, Hsieh ML, Khataokar A, et al. Cyclic di-GMP inhibits Vibrio cholerae motility by repressing induction of transcription and inducing extracellular polysaccharide production. Mol Microbiol. 2013;90:1262–1276. 24134710 10.1111/mmi.12432 PMC3881292

[100] Koestler BJ, Waters CM. Bile acids and bicarbonate inversely regulate intracellular cyclic di-GMP in Vibrio cholerae. Infect Immun. 2014;82:3002–3014. 24799624 10.1128/IAI.01664-14 PMC4097643

[101] Martinez-Wilson HF, Tamayo R, Tischler AD, et al. The vibrio cholerae hybrid sensor kinase VieS contributes to motility and biofilm regulation by altering the cyclic diguanylate level. J Bacteriol. 2008;190:6439–6447. 18676667 10.1128/JB.00541-08 PMC2565995

[102] Davies BW, Bogard RW, Young TS, et al. Coordinated regulation of accessory genetic elements produces cyclic di-nucleotides for V. cholerae virulence. Cell. 2012;149:358–370. 22500802 10.1016/j.cell.2012.01.053 PMC3620040

[103] Severin GB, Ramliden MS, Hawver LA, et al. Direct activation of a phospholipase by cyclic GMP-AMP in El Tor Vibrio cholerae. Proc Natl Acad Sci U S A. 2018;115:E6048–E55. 29891656 10.1073/pnas.1801233115 PMC6042076

[104] Bhattacharyya S, Ghosh S, Shant J, et al. Role of the W07-toxin on Vibrio cholerae-induced diarrhoea. Biochim Biophys Acta. 2004;1670:69–80. 14729143 10.1016/j.bbagen.2003.10.016

[105] Ellis CN, LaRocque RC, Uddin T, et al. Comparative proteomic analysis reveals activation of mucosal innate immune signaling pathways during cholera. Infect Immun. 2015;83:1089–1103. 25561705 10.1128/IAI.02765-14 PMC4333457

[106] Bourque DL, Bhuiyan TR, Genereux DP, et al. Analysis of the human mucosal response to cholera reveals sustained activation of innate immune signaling pathways. Infect Immun. 2018;86:e00594-17. 10.1128/IAI.00594-17 PMC5778365 29133347

[107] Wang H, Chen S, Zhang J, et al. Catalases promote resistance of oxidative stress in Vibrio cholerae. PloS One. 2012;7:e53383. 23300923 10.1371/journal.pone.0053383 PMC3534063

[108] Liu Z, Wang H, Zhou Z, et al. Thiol-based switch mechanism of virulence regulator AphB modulates oxidative stress response in Vibrio cholerae. Mol Microbiol. 2016;102:939–949. 27625149 10.1111/mmi.13524 PMC5123930

[109] Xia X, Larios-Valencia J, Liu Z, et al. OxyR-activated expression of Dps is important for Vibrio cholerae oxidative stress resistance and pathogenesis. PloS One. 2017;12:e0171201. 28151956 10.1371/journal.pone.0171201 PMC5289545

[110] Wholey WY, Jakob U. Hsp33 confers bleach resistance by protecting elongation factor Tu against oxidative degradation in Vibrio cholerae. Mol Microbiol. 2012;83:981–991. 22296329 10.1111/j.1365-2958.2012.07982.x PMC3288485

[111] Wang H, Naseer N, Chen Y, et al. OxyR2 modulates oxyr1 activity and vibrio cholerae oxidative stress response. Infect Immun. 2017;85:e00929-16. 10.1128/IAI.00929-16 PMC5364302 28138024

[112] Goulart CL, Barbosa LC, Bisch PM, et al. Catalases and PhoB/PhoR system independently contribute to oxidative stress resistance in Vibrio cholerae O1. Microbiology. 2016;162:1955–1962. 27665757 10.1099/mic.0.000364

[113] Joelsson A, Kan B, Zhu J. Quorum sensing enhances the stress response in Vibrio cholerae. Appl Environ Microbiol. 2007;73:3742–3746. 17434996 10.1128/AEM.02804-06 PMC1932696

[114] Wang H, Xing X, Wang J, et al. Hypermutation-induced in vivo oxidative stress resistance enhances Vibrio cholerae host adaptation. PLoS Pathog. 2018;14:e1007413. 30376582 10.1371/journal.ppat.1007413 PMC6226196

[115] Winter SE, Winter MG, Xavier MN, et al. Host-derived nitrate boosts growth of E. coli in the inflamed gut. Science. 2013;339:708–711. 23393266 10.1126/science.1232467 PMC4004111

[116] Vazquez-Torres A, Jones-Carson J, Mastroeni P, et al. Antimicrobial actions of the NADPH phagocyte oxidase and inducible nitric oxide synthase in experimental salmonellosis. I. Effects on microbial killing by activated peritoneal macrophages in vitro. J Exp Med. 2000;192:227–236. 10899909 10.1084/jem.192.2.227 PMC2193262

[117] Lopez CA, Winter SE, Rivera-Chavez F, et al. Phage-mediated acquisition of a type III secreted effector protein boosts growth of salmonella by nitrate respiration. mBio. 2012;3:00143. 10.1128/mBio.00143-12 PMC3374392 22691391

[118] Winter SE, Thiennimitr P, Winter MG, et al. Gut inflammation provides a respiratory electron acceptor for Salmonella. Nature. 2010;467:426–429. 20864996 10.1038/nature09415 PMC2946174

[119] Bueno E, Sit B, Waldor MK, et al. Anaerobic nitrate reduction divergently governs population expansion of the enteropathogen Vibrio cholerae. Nat Microbiol. 2018;3:1346–1353. 30275512 10.1038/s41564-018-0253-0 PMC6443258

[120] Stern AM, Hay AJ, Liu Z, et al. The NorR regulon is critical for Vibrio cholerae resistance to nitric oxide and sustained colonization of the intestines. mBio. 2012;3:e00013–12. 22511349 10.1128/mBio.00013-12 PMC3345576

[121] Stern AM, Liu B, Bakken LR, et al. A novel protein protects bacterial iron-dependent metabolism from nitric oxide. J Bacteriol. 2013;195:4702–4708. 23935055 10.1128/JB.00836-13 PMC3807435

[122] Chakrabarti S, Sengupta N, Chowdhury R. Role of DnaK in in vitro and in vivo expression of virulence factors of Vibrio cholerae. Infect Immun. 1999;67:1025–1033. 10024539 10.1128/iai.67.3.1025-1033.1999 PMC96425

[123] Iwanaga M, Yamamoto K, Higa N, et al. Culture conditions for stimulating cholera toxin production by Vibrio cholerae O1 El Tor. Microbiol Immunol. 1986;30:1075–1083. 3543624 10.1111/j.1348-0421.1986.tb03037.x

[124] Lee SH, Hava DL, Waldor MK, et al. Regulation and temporal expression patterns of Vibrio cholerae virulence genes during infection. Cell. 1999;99:625–634. 10612398 10.1016/s0092-8674(00)81551-2

[125] Mandlik A, Livny J, Robins WP, et al. RNA-Seq-based monitoring of infection-linked changes in Vibrio cholerae gene expression. Cell Host Microbe. 2011;10:165–174. 21843873 10.1016/j.chom.2011.07.007 PMC3166260

[126] Hsiao A, Liu Z, Joelsson A, et al. Vibrio cholerae virulence regulator-coordinated evasion of host immunity. Proc Natl Acad Sci U S A. 2006;103:14542–14547. 16983078 10.1073/pnas.0604650103 PMC1599996

[127] Watnick PI, Fullner KJ, Kolter R. A role for the mannose-sensitive hemagglutinin in biofilm formation by Vibrio cholerae El Tor. J Bacteriol. 1999;181:3606–3609. 10348878 10.1128/jb.181.11.3606-3609.1999 PMC93833

[128] Dickinson EC, Gorga JC, Garrett M, et al. Immunoglobulin A supplementation abrogates bacterial translocation and preserves the architecture of the intestinal epithelium. Surgery. 1998;124:284–290. 9706150

[129] Macpherson AJ, Hunziker L, McCoy K, et al. IgA responses in the intestinal mucosa against pathogenic and non-pathogenic microorganisms. Microbes Infect. 2001;3:1021–1035. 11580989 10.1016/s1286-4579(01)01460-5

[130] Royle L, Roos A, Harvey DJ, et al. Secretory IgA N- and O-glycans provide a link between the innate and adaptive immune systems. J Biol Chem. 2003;278:20140–20153. 12637583 10.1074/jbc.M301436200

[131] DiRita VJ, Parsot C, Jander G, et al. Regulatory cascade controls virulence in Vibrio cholerae. Proc Natl Acad Sci USA. 1991;88:5403–5407. 2052618 10.1073/pnas.88.12.5403 PMC51881

[132] Cakar F, Zingl FG, Moisi M, et al. In vivo repressed genes of Vibrio cholerae reveal inverse requirements of an H(+)/Cl(-) transporter along the gastrointestinal passage. Proc Natl Acad Sci U S A. 2018;115:E2376–E85. 29463743 10.1073/pnas.1716973115 PMC5877934

[133] Osorio CG, Crawford JA, Michalski J, et al. Second-generation recombination-based in vivo expression technology for large-scale screening for Vibrio cholerae genes induced during infection of the mouse small intestine. Infect Immun. 2005;73:972–980. 15664940 10.1128/IAI.73.2.972-980.2005 PMC546989

[134] Schild S, Tamayo R, Nelson EJ, et al. Genes induced late in infection increase fitness of vibrio cholerae after release into the environment. Cell Host Microbe. 2007;2:264–277. 18005744 10.1016/j.chom.2007.09.004 PMC2169296

[135] Fuqua C, Winans SC, Greenberg EP. Census and consensus in bacterial ecosystems: the LuxR-LuxI family of quorum-sensing transcriptional regulators. Annu Rev Microbiol. 1996;50:727–751. 8905097 10.1146/annurev.micro.50.1.727

[136] Waters CM, Bassler BL. Quorum sensing: cell-to-cell communication in bacteria. Annu Rev Cell Dev Biol. 2005;21:319–346. 16212498 10.1146/annurev.cellbio.21.012704.131001

[137] Fuqua WC, Winans SC, Greenberg EP. Quorum sensing in bacteria: the LuxR-LuxI family of cell density-responsive transcriptional regulators. J Bacteriol. 1994;176:269–275. 8288518 10.1128/jb.176.2.269-275.1994 PMC205046

[138] Mukherjee S, Bassler BL. Bacterial quorum sensing in complex and dynamically changing environments. Nat Rev Microbiol. 2019;17:371–382. 30944413 10.1038/s41579-019-0186-5 PMC6615036

[139] Lenz DH, Mok KC, Lilley BN, et al. The small RNA chaperone Hfq and multiple small RNAs control quorum sensing in Vibrio harveyi and Vibrio cholerae. Cell. 2004;118:69–82. 15242645 10.1016/j.cell.2004.06.009

[140] Chen X, Schauder S, Potier N, et al. Structural identification of a bacterial quorum-sensing signal containing boron. Nature. 2002;415:545–549. 11823863 10.1038/415545a

[141] Higgins DA, Pomianek ME, Kraml CM, et al. The major Vibrio cholerae autoinducer and its role in virulence factor production. Nature. 2007;450:883–886. 18004304 10.1038/nature06284

[142] Kelly RC, Bolitho ME, Higgins DA, et al. The Vibrio cholerae quorum-sensing autoinducer CAI-1: analysis of the biosynthetic enzyme CqsA. Nat Chem Biol. 2009;5:891–895. 19838203 10.1038/nchembio.237 PMC2847429

[143] Neiditch MB, Federle MJ, Pompeani AJ, et al. Ligand-induced asymmetry in histidine sensor kinase complex regulates quorum sensing. Cell. 2006;126:1095–1108. 16990134 10.1016/j.cell.2006.07.032 PMC3468944

[144] Jung SA, Chapman CA, Ng WL. Quadruple quorum-sensing inputs control Vibrio cholerae virulence and maintain system robustness. PLoS Pathog. 2015;11:e1004837. 25874462 10.1371/journal.ppat.1004837 PMC4398556

[145] Papenfort K, Silpe JE, Schramma KR, et al. A Vibrio cholerae autoinducer-receptor pair that controls biofilm formation. Nat Chem Biol. 2017;13:551–557. 28319101 10.1038/nchembio.2336 PMC5391282

[146] Liu Z, Hsiao A, Joelsson A, et al. The transcriptional regulator VqmA increases expression of the quorum-sensing activator HapR in Vibrio cholerae. J Bacteriol. 2006;188:2446–2453. 16547031 10.1128/JB.188.7.2446-2453.2006 PMC1428415

[147] Teschler JK, Zamorano-Sanchez D, Utada AS, et al. Living in the matrix: assembly and control of Vibrio cholerae biofilms. Nat Rev Microbiol. 2015;13:255–268. 25895940 10.1038/nrmicro3433 PMC4437738

[148] Papenfort K, Forstner KU, Cong JP, et al. Differential RNA-seq of Vibrio cholerae identifies the VqmR small RNA as a regulator of biofilm formation. Proc Natl Acad Sci U S A. 2015;112:E766–75. 25646441 10.1073/pnas.1500203112 PMC4343088

[149] Herzog R, Peschek N, Frohlich KS, et al. Three autoinducer molecules act in concert to control virulence gene expression in Vibrio cholerae. Nucleic Acids Res. 2019;47:3171–3183. 30649554 10.1093/nar/gky1320 PMC6451090

[150] Hammer BK, Bassler BL. Quorum sensing controls biofilm formation in Vibrio cholerae. Mol Microbiol. 2003;50:101–104. 14507367 10.1046/j.1365-2958.2003.03688.x

[151] Vaitkevicius K, Lindmark B, Ou G, et al. A Vibrio cholerae protease needed for killing of Caenorhabditis elegans has a role in protection from natural predator grazing. Proc Natl Acad Sci U S A. 2006;103:9280–9285. 16754867 10.1073/pnas.0601754103 PMC1482601

[152] Colwell RR, Huq A, Islam MS, et al. Reduction of cholera in Bangladeshi villages by simple filtration. Proc Natl Acad Sci U S A. 2003;100:1051–1055. 12529505 10.1073/pnas.0237386100 PMC298724

[153] Liu Z, Miyashiro T, Tsou A, et al. Mucosal penetration primes Vibrio cholerae for host colonization by repressing quorum sensing. Proc Natl Acad Sci U S A. 2008;105:9769–9774. 18606988 10.1073/pnas.0802241105 PMC2474479

[154] Finkelstein RA, Boesman-Finkelstein M, Chang Y, et al. Vibrio cholerae hemagglutinin/protease, colonial variation, virulence, and detachment. Infect Immun. 1992;60:472–478. 1730478 10.1128/iai.60.2.472-478.1992 PMC257651

[155] Nielsen AT, Dolganov NA, Otto G, et al. RpoS controls the Vibrio cholerae mucosal escape response. PLoS Pathog. 2006;2:e109. 17054394 10.1371/journal.ppat.0020109 PMC1617127

[156] Howard MF, Bina XR, Bina JE. Indole inhibits ToxR regulon expression in vibrio cholerae. Infect Immun. 2019;87:e00776-18. 10.1128/IAI.00776-18 PMC6386550 30617203

[157] Bina XR, Taylor DL, Vikram A, et al. Vibrio cholerae ToxR downregulates virulence factor production in response to cyclo(Phe-Pro). mBio. 2013;4:e00366–13. 10.1128/mBio.00366-13 PMC3760244 23982069

[158] Meibom KL, Blokesch M, Dolganov NA, et al. Chitin induces natural competence in Vibrio cholerae. Science. 2005;310:1824–1827. 16357262 10.1126/science.1120096

[159] Tsou AM, Cai T, Liu Z, et al. Regulatory targets of quorum sensing in Vibrio cholerae: evidence for two distinct HapR-binding motifs. Nucleic Acids Res. 2009;37:2747–2756. 19276207 10.1093/nar/gkp121 PMC2677876

[160] Tsou AM, Zhu J. Quorum sensing negatively regulates hemolysin transcriptionally and posttranslationally in Vibrio cholerae. Infect Immun. 2010;78:461–467. 19858311 10.1128/IAI.00590-09 PMC2798175

[161] Silpe JE, Bassler BL. A host-produced quorum-sensing autoinducer controls a phage lysis-lysogeny decision. Cell. 2019;176:268–80e13. 30554875 10.1016/j.cell.2018.10.059 PMC6329655

[162] Wang Y, Wang H, Cui Z, et al. The prevalence of functional quorum-sensing systems in recently emerged vibrio cholerae toxigenic strains. Environ Microbiol Rep. 2011;3:218–222. 21643457 10.1111/j.1758-2229.2010.00212.x PMC3107014

[163] Joelsson A, Liu Z, Zhu J. Genetic and phenotypic diversity of quorum-sensing systems in clinical and environmental isolates of Vibrio cholerae. Infect Immun. 2006;74:1141–1147. 16428762 10.1128/IAI.74.2.1141-1147.2006 PMC1360356

[164] Talyzina NM, Ingvarsson PK, Zhu J, et al. Molecular diversification in the quorum-sensing system of Vibrio cholerae: role of natural selection in the emergence of pandemic strains. Appl Environ Microbiol. 2009;75:3808–3812. 19346342 10.1128/AEM.02496-08 PMC2687267

[165] Katzianer DS, Wang H, Carey RM, et al. “Quorum non-sensing”: social cheating and deception in vibrio cholerae. Appl Environ Microbiol. 2015;81:3856–3862. 25819968 10.1128/AEM.00586-15 PMC4421053

[166] Sender R, Fuchs S, Milo R. Are we really vastly outnumbered? revisiting the ratio of bacterial to host cells in humans. Cell. 2016;164:337–340. 26824647 10.1016/j.cell.2016.01.013

[167] Qin J, Li R, Raes J, et al. A human gut microbial gene catalogue established by metagenomic sequencing. Nature. 2010;464:59–65. 20203603 10.1038/nature08821 PMC3779803

[168] Gorbach SL, Banwell JG, Jacobs B, et al. Intestinal microflora in Asiatic cholera. I. “Rice-water” stool. J Infect Dis. 1970;121:32–37. 4903624 10.1093/infdis/121.1.32

[169] Hsiao A, Ahmed AMS, Subramanian S, et al. Members of the human gut microbiota involved in recovery from Vibrio cholerae infection. Nature. 2014;515:423. 25231861 10.1038/nature13738 PMC4353411

[170] Monira S, Alam NH, Suau A, et al. Time course of bacterial diversity in stool samples of malnourished children with cholera receiving treatment. J Pediatr Gastroenterol Nutr. 2009;48:571–578. 19252449 10.1097/MPG.0b013e3181831867

[171] Monira S, Nakamura S, Gotoh K, et al. Metagenomic profile of gut microbiota in children during cholera and recovery. Gut Pathog. 2013;5:1. 23369162 10.1186/1757-4749-5-1 PMC3574833

[172] Kieser S, Sarker SA, Sakwinska O, et al. Bangladeshi children with acute diarrhoea show faecal microbiomes with increased Streptococcus abundance, irrespective of diarrhoea aetiology. Environ Microbiol. 2018;20:2256–2269. 29786169 10.1111/1462-2920.14274

[173] David LA, Weil A, Ryan ET, et al. Gut microbial succession follows acute secretory diarrhea in humans. mBio. 2015;6:e00381–15. 25991682 10.1128/mBio.00381-15 PMC4442136

[174] Subramanian S, Huq S, Yatsunenko T, et al. Persistent gut microbiota immaturity in malnourished Bangladeshi children. Nature. 2014;510:417. 24896187 10.1038/nature13421 PMC4189846

[175] Gorbach SL, Banwell JG, Jacobs B, et al. Intestinal microflora in Asiatic cholera. II. The small bowel. J Infect Dis. 1970;121:38–45. 4903625 10.1093/infdis/121.1.38

[176] Freter R. Experimental enteric Shigella and Vibrio infections in mice and guinea pigs. J Exp Med. 1956;104:411–418. 13357693 10.1084/jem.104.3.411 PMC2136576

[177] Sack RB, Miller CE. Progressive changes of Vibrio serotypes in germ-free mice infected with Vibrio cholerae. J Bacteriol. 1969;99:688–695. 5370274 10.1128/jb.99.3.688-695.1969 PMC250082

[178] Seedorf H, Griffin NW, Ridaura VK, et al. Bacteria from diverse habitats colonize and compete in the mouse gut. Cell. 2014;159:253–266. 25284151 10.1016/j.cell.2014.09.008 PMC4194163

[179] Midani FS, Weil AA, Chowdhury F, et al. Human gut microbiota predicts susceptibility to vibrio cholerae infection. J Infect Dis. 2018;218:645–653. 29659916 10.1093/infdis/jiy192 PMC6047457

[180] Alavi S, Mitchell JD, Cho JY, et al. Interpersonal gut microbiome variation drives susceptibility and resistance to cholera infection. Cell. 2020;181:1533–46.e13. 32631492 10.1016/j.cell.2020.05.036 PMC7394201

[181] Watve S, Barrasso K, Jung SA, et al. Parallel quorum-sensing system in Vibrio cholerae prevents signal interference inside the host. PLoS Pathog. 2020;16:e1008313. 32059031 10.1371/journal.ppat.1008313 PMC7046293

[182] Sun J, Daniel R, Wagner-Dobler I, et al. Is autoinducer-2 a universal signal for interspecies communication: a comparative genomic and phylogenetic analysis of the synthesis and signal transduction pathways. BMC Evol Biol. 2004;4:36. 15456522 10.1186/1471-2148-4-36 PMC524169

[183] Miller ST, Xavier KB, Campagna SR, et al. Salmonella typhimurium recognizes a chemically distinct form of the bacterial quorum-sensing signal AI-2. Mol Cell. 2004;15:677–687. 15350213 10.1016/j.molcel.2004.07.020

[184] Xavier KB, Bassler BL. Interference with AI-2-mediated bacterial cell-cell communication. Nature. 2005;437:750–753. 16193054 10.1038/nature03960 PMC1388276

[185] Chand D, Avinash VS, Yadav Y, et al. Molecular features of bile salt hydrolases and relevance in human health. Biochim Biophys Acta Gen Subj. 2017;1861:2981–2991. 27681686 10.1016/j.bbagen.2016.09.024

[186] De Smet I, Van Hoorde L, Vande Woestyne M, et al. Significance of bile salt hydrolytic activities of lactobacilli. J Appl Bacteriol. 1995;79:292–301. 7592123 10.1111/j.1365-2672.1995.tb03140.x

[187] Grill JP, Cayuela C, Antoine JM, et al. Isolation and characterization of a Lactobacillus amylovorus mutant depleted in conjugated bile salt hydrolase activity: relation between activity and bile salt resistance. J Appl Microbiol. 2000;89:553–563. 11054157 10.1046/j.1365-2672.2000.01147.x

[188] Sayin SI, Wahlstrom A, Felin J, et al. Gut microbiota regulates bile acid metabolism by reducing the levels of tauro-beta-muricholic acid, a naturally occurring FXR antagonist. Cell Metab. 2013;17:225–235. 23395169 10.1016/j.cmet.2013.01.003

[189] Duan F, March JC. Engineered bacterial communication prevents Vibrio cholerae virulence in an infant mouse model. Proc Natl Acad Sci U S A. 2010;107:11260–11264. 20534565 10.1073/pnas.1001294107 PMC2895089

[190] Mao N, Cubillos-Ruiz A, Cameron DE, et al. Probiotic strains detect and suppress cholera in mice. Sci Transl Med. 2018;10:eaao2586. 10.1126/scitranslmed.aao2586 PMC7821980 29899022

[191] Hubbard TP, Billings G, Dorr T, et al. A live vaccine rapidly protects against cholera in an infant rabbit model. Sci Transl Med. 2018;10:eaap8423. 10.1126/scitranslmed.aap8423 PMC6500431 29899024

[192] McNulty NP, Yatsunenko T, Hsiao A, et al. The impact of a consortium of fermented milk strains on the gut microbiome of gnotobiotic mice and monozygotic twins. Sci Transl Med. 2011;3:106ra. 10.1126/scitranslmed.3002701 PMC3303609 22030749

[193] MacIntyre DL, Miyata ST, Kitaoka M, et al. The Vibrio cholerae type VI secretion system displays antimicrobial properties. Proc Natl Acad Sci U S A. 2010;107:19520–19524. 20974937 10.1073/pnas.1012931107 PMC2984155

[194] Pukatzki S, Ma AT, Sturtevant D, et al. Identification of a conserved bacterial protein secretion system in Vibrio cholerae using the Dictyostelium host model system. Proc Natl Acad Sci U S A. 2006;103:1528–1533. 16432199 10.1073/pnas.0510322103 PMC1345711

[195] Verster AJ, Ross BD, Radey MC, et al. The landscape of type VI secretion across human gut microbiomes reveals its role in community composition. Cell Host Microbe. 2017;22:411–9e4. 28910638 10.1016/j.chom.2017.08.010 PMC5679258

[196] Wexler AG, Bao Y, Whitney JC, et al. Human symbionts inject and neutralize antibacterial toxins to persist in the gut. Proc Natl Acad Sci U S A. 2016;113:3639–3644. 26957597 10.1073/pnas.1525637113 PMC4822603

[197] Fu Y, Waldor MK, Mekalanos JJ. Tn-Seq analysis of Vibrio cholerae intestinal colonization reveals a role for T6SS-mediated antibacterial activity in the host. Cell Host Microbe. 2013;14:652–663. 24331463 10.1016/j.chom.2013.11.001 PMC3951154

[198] Fast D, Kostiuk B, Foley E, et al. Commensal pathogen competition impacts host viability. Proc Natl Acad Sci U S A. 2018;115:7099–7104. 29915049 10.1073/pnas.1802165115 PMC6142279

[199] Zhao W, Caro F, Robins W, et al. Antagonism toward the intestinal microbiota and its effect on Vibrio cholerae virulence. Science. 2018;359:210–213. 29326272 10.1126/science.aap8775 PMC8010019

[200] Logan SL, Thomas J, Yan J, et al. The Vibrio cholerae type VI secretion system can modulate host intestinal mechanics to displace gut bacterial symbionts. Proc Natl Acad Sci U S A. 2018;115:E3779–E87. 29610339 10.1073/pnas.1720133115 PMC5910850

[201] Zheng J, Shin OS, Cameron DE, et al. Quorum sensing and a global regulator TsrA control expression of type VI secretion and virulence in Vibrio cholerae. Proc Natl Acad Sci U S A. 2010;107:21128–21133. 21084635 10.1073/pnas.1014998107 PMC3000250

[202] Watve SS, Thomas J, Hammer BK. CytR is a global positive regulator of competence, type vi secretion, and chitinases in vibrio cholerae. PloS One. 2015;10:e0138834. 26401962 10.1371/journal.pone.0138834 PMC4581735

[203] Shao Y, Bassler BL. Quorum regulatory small RNAs repress type VI secretion in Vibrio cholerae. Mol Microbiol. 2014;92:921–930. 24698180 10.1111/mmi.12599 PMC4038675

[204] Bachmann V, Kostiuk B, Unterweger D, et al. Bile salts modulate the mucin-activated Type VI secretion system of pandemic vibrio cholerae. PLoS Negl Trop Dis. 2015;9:e0004031. 26317760 10.1371/journal.pntd.0004031 PMC4552747

[205] Sicard JF, Le Bihan G, Vogeleer P, et al. Interactions of intestinal bacteria with components of the intestinal mucus. Front Cell Infect Microbiol. 2017;7:387. 28929087 10.3389/fcimb.2017.00387 PMC5591952

[206] Li H, Limenitakis JP, Fuhrer T, et al. The outer mucus layer hosts a distinct intestinal microbial niche. Nat Commun. 2015;6:8292. 26392213 10.1038/ncomms9292 PMC4595636

[207] Derrien M, Vaughan EE, Plugge CM, et al. Akkermansia muciniphila gen. nov., sp. nov., a human intestinal mucin-degrading bacterium. Int J Syst Evol Microbiol. 2004;54:1469–1476. 15388697 10.1099/ijs.0.02873-0

[208] Verma J, Bag S, Saha B, et al. Genomic plasticity associated with antimicrobial resistance in Vibrio cholerae. Proc Natl Acad Sci U S A. 2019;116:6226–6231. 30867296 10.1073/pnas.1900141116 PMC6442563

[209] Faith JJ, Ahern PP, Ridaura VK, et al. Identifying gut microbe-host phenotype relationships using combinatorial communities in gnotobiotic mice. Sci Transl Med. 2014;6:220ra11. 10.1126/scitranslmed.3008051 PMC3973144 24452263

[210] Claesson MJ, Jeffery IB, Conde S, et al. Gut microbiota composition correlates with diet and health in the elderly. Nature. 2012;488:178–184. 22797518 10.1038/nature11319

[211] Faith JJ, McNulty NP, Rey FE, et al. Predicting a human gut microbiota’s response to diet in gnotobiotic mice. Science. 2011;333:101–104. 21596954 10.1126/science.1206025 PMC3303606

[212] Smith MI, Yatsunenko T, Manary MJ, et al. Gut microbiomes of Malawian twin pairs discordant for kwashiorkor. Science. 2013;339:548–554. 23363771 10.1126/science.1229000 PMC3667500

[213] Zimmer J, Lange B, Frick JS, et al. A vegan or vegetarian diet substantially alters the human colonic faecal microbiota. Eur J Clin Nutr. 2012;66:53–60. 21811294 10.1038/ejcn.2011.141

